# Characterisation of a natural variant of the γ-butyrolactone signalling receptor

**DOI:** 10.1186/1756-0500-5-379

**Published:** 2012-07-27

**Authors:** Marco Gottelt, Andrew Hesketh, Robert Bunet, Pranav Puri, Eriko Takano

**Affiliations:** 1 Department of Microbial Physiology, Groningen Biomolecular Sciences and Biotechnology Institute, University of Groningen, Nijenborgh 7, 9747 AG, Groningen, The Netherlands; 2 Present address: Life Technologies, Frankfurter Str. 129b, 64293, Darmstadt, Germany; 3 Department of Molecular Microbiology, John Innes Centre, Norwich Research Park, Colney, Norwich, NR4 7UH, UK; 4 Present address: Department of Biochemistry, New Building (Sanger), 80 Tennis Court Road, Old Addenbrooke's Site, Cambridge, CB2 1GA, UK; 5 Present address: Équipe de Biologie Moléculaire Marine - PROTEE, Université du Sud Toulon-Var, BP 20132, Avenue de l'Université, 83957, La Garde Cedex, France; 6 Present address: Department of Enzymology, Groningen Biomolecular Sciences and Biotechnology Institute, University of Groningen, Nijenborgh 4, 9747 AG, Groningen, The Netherlands

**Keywords:** Gamma-butyrolactone receptor, Gene regulation, Antibiotic production, *Streptomyces*, ScbR, M600

## Abstract

**Background:**

The control of antibiotic production in *Streptomyces coelicolor* A3(2) involves complicated regulatory networks with multiple regulators controlling the expression of antibiotic biosynthetic pathways. One such regulatory network is that of the γ-butyrolactones, the so-called *S. coelicolor* butanolide (SCB) system. The γ-butyrolactones in this system serve as signalling molecules and bind to the receptor protein ScbR, releasing the repression of its target genes. The resulting expression changes affect the production of the two pigmented antibiotics Act and Red, as well as the transcription of the *cpk* antibiotic biosynthesis gene cluster and the synthesis of the γ-butyrolactones themselves.

**Results:**

We identified a natural variant of ScbR in *S. coelicolor* (ScbR_M600_) that differs from ScbR in the genome-sequenced strain M145 (ScbR_M145_) by a single amino acid change, R120S. ScbR_M600_ is impaired in its DNA binding ability and alters the expression of the pathway-specific regulatory genes of the *red* and *cpk* antibiotic biosynthesis gene clusters. Also, expression of the γ-butyrolactone biosynthesis gene *scbA* and production of the signalling molecules is slightly reduced.

**Conclusions:**

The γ-butyrolactone receptor, ScbR, plays a key role in the SCB regulatory cascade and in determining the onset of the expression of the antibiotic regulatory genes.

## Background

Streptomycetes show a complex morphological differentiation and produce a vast variety of secondary metabolites with great value in the pharmaceutical, chemical and agricultural industries [1,2]. The genome sequence of the model streptomycete *Streptomyces coelicolor* A3(2) strain M145, has been determined and is publicly available [3]. *S. coelicolor* A3(2) strains M145 and M600 are two of many strains independently derived from *S. coelicolor* A3(2). Both are prototrophic plasmid-free derivatives, but M145 was derived using both mutagenesis and recombination while creation of M600 did not involve any mutagenesis [4]. Genetically, M600 differs from M145 in that it possesses long terminal inverted repeats (TIRs) at both ends of the chromosome, resulting in the duplication of 1005 genes compared to M145. This does not, however, appear to significantly affect total expression of the duplicated genes, since highly similar transcript levels could be observed when comparing the two strains [4].

In several *Streptomyces* species, small autoregulatory molecules called γ-butyrolactones are involved in controlling the onset of secondary metabolite production and morphological differentiation (reviewed in [5]). There are numerous diverse and complex regulatory systems involving γ-butyrolactones with the signalling cascade for A-factor in *S. griseus* being the best studied [6,7]. In *S. coelicolor*, γ-butyrolactones stimulating the production of Act and Red have been identified [8], together with the genes involved in γ-butyrolactone synthesis (*scbA*) and γ-butyrolactone binding (*scbR*). ScbR regulates transcription of both *scbA* and itself by binding to the divergent promoter region controlling both genes, and the γ-butyrolactone SCB1 inhibits this binding [9]. The regulatory influence of ScbR has been characterised by DNA microarray analysis, and a role in directly regulating a cryptic Type I polyketide biosynthetic gene cluster (*cpk* cluster) by binding to the promoter of its pathway-specific regulator *cpkO* was identified [10,11]. We recently reported two metabolites derived from the hitherto orphan *cpk* biosynthetic pathway, the yellow pigment yCPK and an antibiotic compound, abCPK [12]. ScbR does not, however, bind to the promoter regions of the pathway-specific regulatory genes for Act and Red synthesis [9], and it appears that SCB1 and *scbAR* do not regulate the production of these antibiotics directly. Nevertheless, an M145Δ*scbR* mutant (M752) is delayed in the production of Act and Red [9].

ScbR is a member of the TetR protein family [13], in which the *Streptomyces* γ-butyrolactone receptors show significant similarity to each other (30–40 % amino acid sequence identity). The crystal structure of a ScbR paralogue in *S. coelicolor*, CprB, has been determined and is assumed to generally represent the structure of γ-butyrolactone receptors [14]. Active as homodimers, members of the TetR family bind to highly specific DNA binding sites in the promoter region of their target genes and typically repress their transcription. The regulatory region of the dimeric regulator contains one independent ligand binding pocket in each subunit. One γ-butyrolactone receptor thus binds two ligand molecules. Binding of γ-butyrolactones causes conformational changes and DNA binding is relieved [14].

In this study, the *S. coelicolor* γ-butyrolactone receptor ScbR in strain M600 (ScbR _M600_) was found to differ from that in the sequenced strain M145 (ScbR_M145_) by a single amino acid change. The effect of the M600-type protein on the production of pigmented antibiotics Act Red, and the yellow compound, yCPK, as well as the γ-butyrolactones was assessed *in vivo*. In addition, the influence of SCBR _M600_ on the expression of genes involved in the butanolide system and secondary metabolism was evaluated by quantitative real time PCR. The effect onDNA and/or γ-butyrolactone binding ability of ScbR due to the amino acid substitution was also investigated *in vivo* and *in vitro.* Sequence analysis was used to determine the prevalence of the two forms of ScbR among strains of *S. coelicolor* and *S. lividans*. The ScbR_M600_ variant is present in only two, independent strains of *S. coelicolor* and its decreased DNA binding activity results in a delay in the transcription of the antibiotic regulatory genes.

## Results

### Two forms of the γ-butyrolactone receptor ScbR in strains of *S. coelicolor*

**Figure 1:**
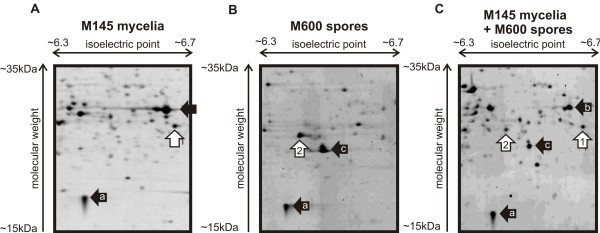
** 2D gel analysis of*S. coelicolor*protein extracts showing the presence of two distinct forms of ScbR in strains M145 and M600.** A.B. C. A part of the gels is shown with separation horizontally by isoelectric point and vertically by molecular weight. Numbered white arrows indicate ScbR from M145 (1) and M600 (2), while black arrows highlight landmark spots (a, b, c) known to be the same in at least one of the other gels.The gels shown in A and B are representative of the results from the analysis of at least two biological and three technical replicate experiments. Panel C is a representative image chosen from two technical replicates.

2D gel-based proteomic analysis of transition phase liquid cultures from* S. coelicolor* strain M145 identified ScbR at a position consistent with its theoretical molecular weight and isoelectric point (spot 1 in Figure 1A). However, in an extensive analysis of strain M600 grown under the same conditions ScbR was never detected (data not shown). In an analysis of protein extracts prepared from spores of M600, ScbR was detected (spot 2 in Figure 1B) but at coordinates corresponding to a significantly more acidic isoelectric point compared to that observed in M145. This difference was confirmed by performing a separation of an equal mixture of the M600 spore extract and the M145 transition phase mycelial extract (Figure 1C), and indicates the occurrence of a modified form of ScbR in *S. coelicolor* strain M600.

### ScbR_M600_ from *S. coelicolor* M600 carries a single amino acid change, R120S, compared to ScbR_M145_ from strain M145

**Figure 2:**
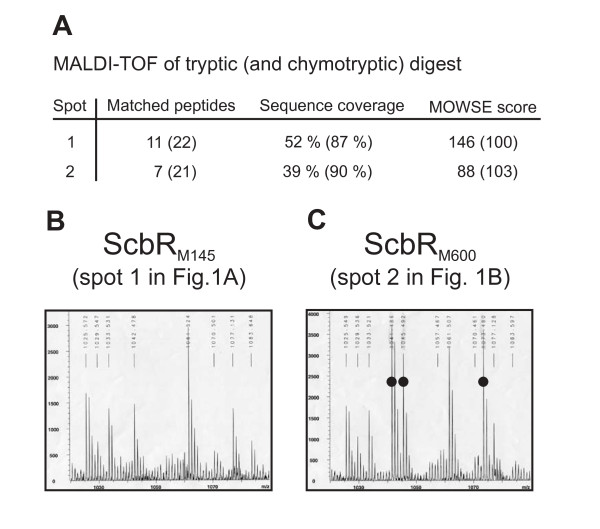
** MALDI-TOF peptide mass fingerprint analysis of *S. coelicolor* strains M145 and M600 protein extracts. A**. Identification of ScbR spots 1 and 2 by mass spectrometry after tryptic and chymotryptic digest. Data from the chymotryptic digests are given in brackets. **B**. **C**. Detail of the 1020 Da to 1090 Da mass range showing three extra peptides in ScbRM600 (spot 2 in A) (at 1041.486, 1045.492 and 1073.480 Da, marked with circles). The extra peak at 1041.486 Da was identified as RSWHETLL by Q-TOF mass spectrometry, and the other peaks are believed to correspond to the same peptide but with the tryptophan modified to kyneurenine (1045.492 Da) and formylkyneurenine (1073.480 Da).

**Figure 3:**
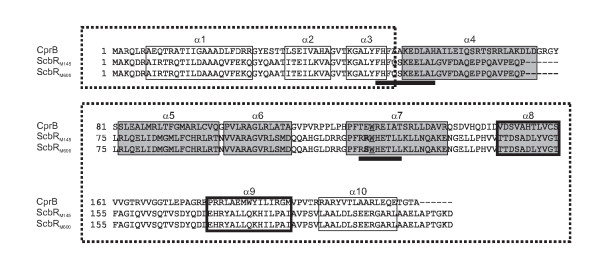
** Amino acid sequence alignment and deduction of functional domains for residues of CprB, ScbR**_**M145**_**and ScbR**_**M600**_**.** The deduction of functional domains and residues is based on CprB data from [14]. The amino acid sequences represent monomers of each protein. Peptides readily detected by MALDI-TOF analysis in ScbR_M145_ (spot 1 in Figure 1A) but not in ScbR_M600_ (spot 2 in Figure 1B) are indicated by black bars (ScbR amino acids 48–58 and 119–126). Residues forming α-helices are boxed and labelled. Boxes are shaded for α-helices 4–8 involved in the formation of the γ-butyrolactone binding pocket. Bold boxes for α-helices 8 and 9 indicate their role in dimerization. Dashed boxes mark the DNA binding domain (α1-3) with α2 and α3 forming a helix-turn-helix motif, and the regulatory domain (α5-10). A highly conserved tryptophan residue directly involved in ligand binding (W127 in CprB; W121 in ScbR) is underlined. The mutated amino acid residue 120 is indicated in bold for ScbR_M145_ (R120) and for ScbR_M600_ (S120).

To identify the M600 ScbR modification, the chymotryptic peptides detected for the ScbR proteins in M145 spot 1 and M600 spot 2 in Figure 1 were compared (Figure 2A,B,C, Additional file 1). Peptides corresponding to all the ScbR amino acid sequence except RRWHETLL and FHFQSKEELAL (indicated by black bars in Figure 3), were detected in the ScbR spot from M600 (Additional file 1). Peptides containing tryptophan can produce up to four peptide peaks in MALDI-TOF analysis due to two successive oxidations of the tryptophan residue (i.e. parent mass +16.0 Da and +32.0 Da), followed by spontaneous deformylation to kyneurenine (parent mass +4.0 Da) [15], and peaks at 1126.62 Da and 1142.62 Da in the data for the M145 spot correspond to the oxidised forms of the parent ion detected at 1110.62 Da. Interestingly, these three peptides are absent in the peptide mass fingerprint for the M600 ScbR spot, but three new peptide peaks apparently corresponding to a tryptophan-containing peptide can be detected at 1041.48 Da, 1045.49 Da (parent +4.0 Da), and 1073.48 Da (parent +32.0 Da; Figure 2B,C). ScbR contains only one tryptophan residue, and the data therefore indicate that in M600 spot 2 ScbR has been modified on the RRWHETLL peptide resulting in a surprising mass loss of 69.09 Da (and an acidic shift in the isoelectric point value of the protein). This data correspond to the replacement of an arginine residue with a serine (69.069 Da). Analysis of the parent peptide ion at 1041.48 Da using Q-TOF mass spectrometry confirmed the sequence of this peptide as RSWHETLL (data not shown). This has also subsequently been verified via sequencing of the *scbR* gene in strain M600 (see below). The M600-type protein (ScbR_M600_) therefore contains a single amino acid change (R120S) compared to the M145-type protein (ScbR_M145_) (Figure 3).

### ScbR_M145_ is the predominant form in *S. coelicolor*

To survey the distribution of the dimorphism identified in ScbR, part of the *scbR* coding region of 16 *S. coelicolor* strains was amplified by PCR using primers ScbRrt1 and ScbRrt2 and the sequence was determined. In addition, to confirm the *scbR* sequence in *S. coelicolor* M600 and *S. lividans* 1326, the entire *scbR* coding sequence and promoter region was amplified using primers ScbR2 and ETS10 (all primers used are listed in Additional file 2).The*scbR* in M600, a single base pair change, c358a, was identified, confirming the amino acid mutation R120S observed in the proteomics analysis. The same mutation was also found in *S. coelicolor* strain A(3)2 N2, but was absent in the other 14 *S. coelicolor* strains tested, which all possessed the M145 genotype (Table 1). No other variants of *scbR* were observed in the strains tested. The *scbR* homologue in *S. lividans* differs from *scbR*_M145_ only by two silent point mutations, g402a and g582t. Thus, the *S. lividans* ScbR amino acid sequence is identical to that of ScbR_M145_. A multi-sequence alignment of the ScbR homologues are shown in Additional file 3. The *scbR* promoter regions from 251 bp upstream of *scbR,* covering the ScbR binding sites of the intergenic region between divergently transcribed *scbA* and *scbR*, are identical in *S. coelicolor* strains M145, M600 and also in the *scbR* homologue of* S. lividans* 1326 (data not shown). Our sequencing data were confirmed by the publicly available genome sequence of *S. lividans* (http://www.broadinstitute.org/annotation/genome/streptomyces_group/Regions.html).

**Table 1:** Bacterial strains used in this study

**Name**	**Description**	***scbR*** **type**	**Reference**
***Escherichia coli***			
JM101	*supEthi* Δ(*lac*-*proAB*) F´ [*traD*36 *proAB*^+^*lacI*^q^*lacZ* ΔM15]	-	[16]
ET12567	Non-methylating strain used for conjugation with *Streptomyces*	-	[17]
***Streptomyces coelicolor*** **A3(2)**			
11	*pheA1*	*scbR*_M145_	[4]
13	*uraA1*	*scbR*_*M145*_	[4]
210	*ura-3*	*scbR*_*M145*_	[4]
290	*cys-4*	*scbR*_*M145*_	[4]
380	*glu-3*	*scbR*_*M145*_	[4]
505	*cysC3*	*scbR*_*M145*_	[4]
A3(2) N1	A3(2) isolate	*scbR*_*M145*_	[4]
A3(2) N2	A3(2) isolate	*scbR*_*M600*_	[4]
A3(2) N3	A3(2) isolate	*scbR*_*M145*_	[4]
A3(2)-Stanford	A3(2) isolate	*scbR*_*M145*_	[4]
CH999	*proA1 argA1 redD60* Δ*act::ermE* SCP1^-^ SCP2^-^	*scbR*_*M145*_	[4]
J1501	*hisA1 uraA1 strA1 pgl-1* SCP1^-^ SCP2^-^	*scbR*_*M145*_	[18]
LW33	M752 + *scbR*_M600_	*scbR*_*M600*_	This study
LW34	M752 + *scbR*_M145_	*scbR*_*M145*_	This study
M132	*pheA1* SCP1^-^ SCP2^-^	*scbR*_*M145*_	[4]
M145	SCP1^-^ SCP2^-^	*scbR*_*M145*_	[19]
M600	SCP1^-^ SCP2^-^	*scbR*_M600_	[20]
M752	M145 Δ*scbR*	*-*	[9]
W3443	wild type	*scbR*_*M145*_	[4]
***Streptomyces lividans***			
1326	wild type	*scbR*_*M145*_ homologue	[18]

### *In vivo* analysis of the *scbR*_M600_ point mutation in a M145 background

Production of pigmented antibiotics in strains M145 and M600 differ, with M600 being notably delayed in the onset of Act and Red biosynthesis, and this is comparable to the phenotypic change observed in strain M145 following deletion of *scbR*[9]. To determine whether the M600 phenotype may be attributed to the point mutation observed in *scbR*_M600_, a M145 ScbR deletion mutant was genetically complemented with constructs expressing either *scbR*_M145_ or *scbR*_M600_ under the native *scbR* promoter.

**Figure 4:**
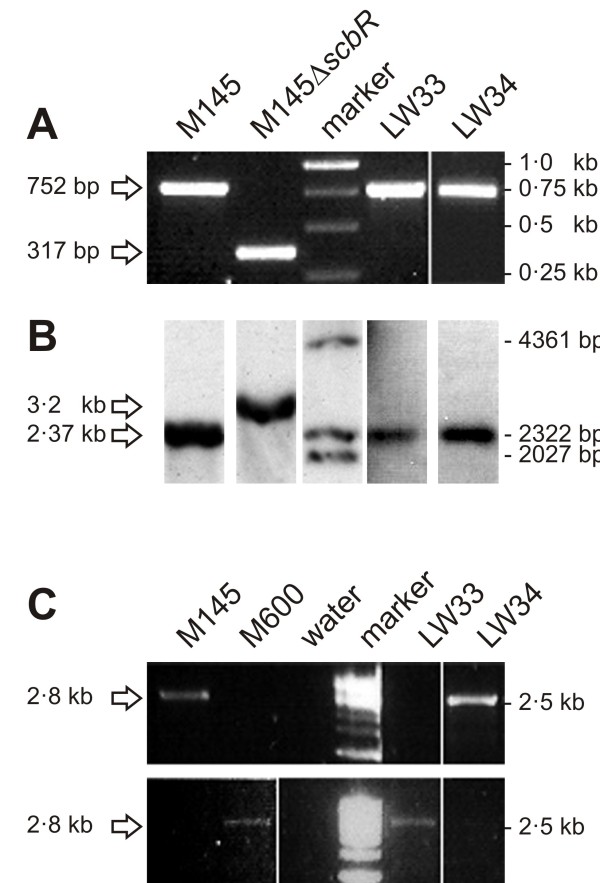
** Verification of full-length*scbR***_**M145/M600**_**in*S. coelicolor*strains LW34 and LW33. A**. PCR was carried out using primers ETseq3 and ETS7 to amplify a 752 bp full-length *scbR*_M145/M600_ fragment and a 317 bp fragment from the Δ*scbR* region. The 317 bp PCR product was only found with the LW33/LW34 parental M145Δ*scbR* mutant. A full-length *scbR*_*M145/M600*_fragment was amplified from a wt control (M145) and from LW33 and LW34. Obtained PCR products are indicated with arrows on the left; sizes of the marker bands are given on the right. The M145-/M600-type of the gene was confirmed by DNA sequence analysis (data not shown). **B**. Southern analysis was carried out using *Nco*I digested genomic DNA of the four strains. A DIG-labelled DNA probe was used to detect the expected 3.2 kb fragment with M145Δ*scbR* and 2.37 kb fragments with the wt control (M145) and LW33 and LW34. Detected DNA fragments are indicated with arrows on the left; sizes of the marker bands are given on the right. **C**. PCR was carried out using primers RCseq31 and scbR-M145_c358 (scbR-M600_c358a) to amplify a 2.8 kb *scbR*_M145_ (*scbR*_M600_)fragment. The latter are specific for the base change between the two *scbR* variants. A *scbR*_M145_ fragment was only obtained with control strain M145 and with LW34, whereas control strain M600 and LW33 showed a *scbR*_M600_ specific PCR product. Using water as template did not give any product. Obtained PCR products are labelled with arrows on the left; a 2.5 kb marker band is indicated on the right.

Plasmids pTE212 and pTE214 carrying *scbR*_M600_ and *scbR*_M145_, respectively, were used to replace the mutated *scbR*_M145_ locus in the M145Δ*scbR*_M145_ in-frame deletion mutant strain M752 [9] to give strains LW33 and LW34. LW33 therefore encodes ScbR_M600_ in the M145 genetic background, and LW34 is the congenic control strain expressing ScbR_M145_. Correct construction of the strains was verified using PCR and Southern analysis (Figure 4A,B). Sequence analysis revealed a silent point mutation, c636t (leading to an “act” triplet instead of “acc”, both resulting in Thr), in *scbR*_M145_ of strain LW34 (pTE214). Besides this mutation, strains LW33 and LW34 only differ by c358a in *scbR*.

Strains LW33 and LW34 were grown in liquid SMM. Samples for RNA and protein isolation, as well as for γ-butyrolactone and antibiotic analysis, were collected at different phases of growth (Table 2).

**Table 2:** Growth and antibiotic production of LW34 and LW33

**time point**		**Growth Curve 1 (GC 1)**				**Growth Curve 2 (GC 2)**			
		**1**	**2**	**3**	**4**	**1**	**2**	**3**	**4**
**growth phase**		**eT**	**mT**	**lT**	**S**	**eT**	**mT**	**lT**	**S**
**OD**_**450**_**(hours)**	**LW34**	1.00 (21)	1.21 (23)	1.26 (24·5)	1.45 (43)	1.25 (18)	1.49 (20)	1.62 (22)	1.92 (40)
	**LW33**	1.04 (21)	1.18 (23)	1.25 (24·5)	1.42 (43)	1.56 (18)	1.38 (20)	1.58 (21·5)	1.23 (40)
**Red**	**LW34**	0.3	0.6	0.9	3.9	0.1	0.2	0.6	0.7
	**LW33**	0.5	0.7	1.0	3.7	0.0	0.2	1.1	2.1
**Act**	**LW34**	0.0	0.0	0.0	1.0	0.2	0.1	0.1	0.5
	**LW33**	0.0	0.0	0.0	1.0	0.1	0.1	0.1	0.7

*S. coelicolor* LW34 and LW33 were grown *in duplo* in liquid SMM (growth curves (GC) 1 and 2). Samples were taken at different phases of growth (early, mid and late transition (eT, mT, lT), and stationary (S) phase). The sampling time is given in hours of growth in brackets underneath the determined OD_450_. Production of actinorhodin (Act) and (undecyl-) prodiginines (Red) is given in μg /dry cell weight mg^-1.^

### Antibiotic production in *S. coelicolor* LW34 and LW33 is comparable

No difference could be determined for Act production. This was consistent between two independent growth experiments (Table 2). For Red, production increased 2-fold in LW33 for GC 2 but the increase was not seen in GC 1 (Table 2).

**Figure 5:**
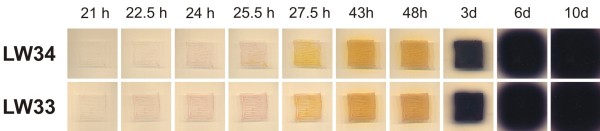
** Secondary metabolite production in solid culture.***S. coelicolor* strains LW34 (*scbR*_M145_) and LW33 (*scbR*_M600_) were incubated on glutamate-supplemented Difco Nutrient agar at 30 °C. Production of pigmented secondary metabolites was followed at 21, 22.5, 24, 25.5, 27.5, 43, 48 h and 3, 6, 10 days.

We recently identified a yellow pigment (yCPK) and an antibiotic compound (abCPK) as metabolites of the hitherto orphan *cpk* gene cluster [12] that may be the primary target of the *S. coelicolor* butanolide system. Therefore, in addition to Act and Red, we compared the production of the yellow compound in LW33 and LW34 grown on solid Difco Nutrient agar supplemented with glutamate where yCPK was found to be produced in high yields by the parental strain M145 [12]. The two tested strains showed no obvious difference in the production of all three pigmented secondary metabolites during ten days of growth (Figure 5). From all these data there seems to be no significant difference in antibiotic production between LW33 and LW34 under the conditions tested, demonstrating that the difference in ScbR isoform does not explain the phenotypic difference of M145 and M600.

### γ-butyrolactone production is slightly delayed in LW33

**Figure 6:**
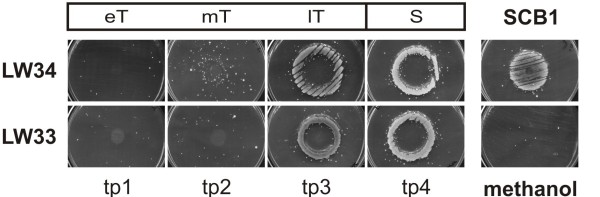
** γ-Butyrolactone production in liquid*S. coelicolor*cultures using the kanamycin bioassay.** The kanamycin bioassay [21] was used to detect γ-butyrolactone production in *S. coelicolor* in liquid SMM at four time points (tp 1–4) during different phases of growth indicated with eT, mT, lT and S (early, mid, late transition, and stationary phase; also see Table 2, GC 2). Growth of a bioassay indicator strain on kanamycin supplemented medium is shown. Kanamycin resistance and thus growth is induced by the presence of γ-butyrolactones. Extracts from strains LW34 and LW33, containing ScbR_M145_ and ScbR_M600_, respectively, resembled each other in the induction of growth, however, with LW34 sparse growth seems to be induced already at time point 2 and the area of growth is increased compared to LW33 with the late transition phase sample. Chemically synthesised *S. coelicolor* γ-butyrolactone (SCB1) was used as positive, the solvent methanol as negative control.

**Figure 7:**
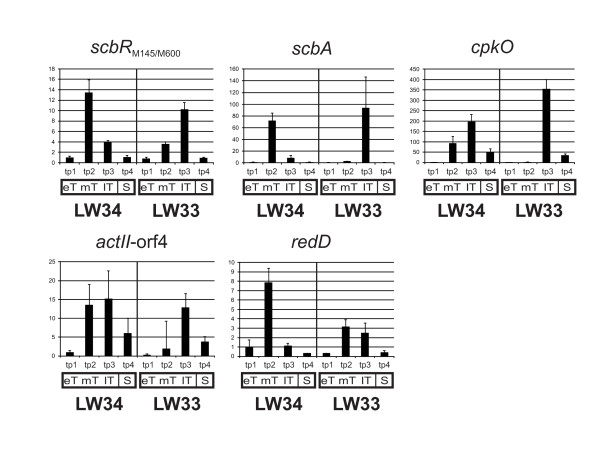
** Transcriptional analysis of*redD*,*actII*-orf4,*cpkO*,*scbA*and*scbA*and*scbR***_**M145/M600**_**using qRT-PCR.** qRT-PCR analysis of the transcription of *redD*, *actII*-orf4, *cpkO*, *scbA* and *scbR*_M145/M600_ using cDNA synthesized from RNA isolated from liquid SMM cultures of *S. coelicolor*LW34 (*scbR*_M145_) and LW33 (*scbR*_M600_). Samples were taken at four time points (tp 1–4) during different phases of growth indicated with eT, mT, lT and S (early, mid, late transition, Sand stationary phase). Gene expression is shown as fold-change relative to the LW34 time point 1 early transition phase sample. Error bars indicate the standard deviation of each three technical replicates. For numerical data see Additional file 4A and 4B.

γ-butyrolactone production of *S. coelicolor* strains LW34 and LW33 was determined using a kanamycin bioassay [21] at different phases of growth (Table 2, GC 2) corresponding to the sampling time points in the transcription analysis. Kanamycin resistance of a *Streptomyces* indicator strain is induced by the presence of γ-butyrolactones in extracts from the tested strains, and the extent of growth of the indicator strain on media containing kanamycin reflects the amount of γ-butyrolactones produced [21]. Extracts from stationary phase cultures of LW33 and LW34 produced similar halos of growth, indicating the presence of similar levels of γ-butyrolactones. Slightly higher levels were detected withmid and late transition phase samples in strain LW34 (Figure 6). This would indicate that ScbR_M600_ in strain LW33 leads to a minor reduction and delay in γ-butyrolactone production compared to LW34, and is consistent with the delay in *scbA* transcription observed in LW33 as shown below (Figure 7).

### Expression of *scbR*, *scbA*, *redD* and *cpkO* is altered in strain LW33 containing the mutant ScbR_M600_

Quantitative real time PCR (qRT-PCR) was used to measure the expression levels of the pathway-specific regulators of the *act* (*actII*-ORF4), *red* (*redD*) and the *cpk* (*cpkO*) antibiotic biosynthesis gene clusters, and of *scbA* and *scbR* of the *S. coelicolor* butanolide system in the presence of the two forms of ScbR (Figure 7). Compared to the control strain LW34, *redD* expression was 2.5-fold lower at mid transition phase (mT) in LW33 harbouring the ScbR_M600_. *RedD* expression did not change dramatically from mid transition to late transition phase in LW33, while in LW34, the expression dramatically decreased. This resulted in a >2-fold higher*redD* expression in LW33 compared to LW34 at late transition phase. For GC 2, The onset of *actII*-ORF4 transcription was delayed in LW33 but this was not observed in GC 1. Expression of *cpkO* was only transcribed in late transition phase (lT), and thus later than in LW34. Also, the onset of *scbA*transcription was delayed in LW33 and shifted from mid to late transition. Also with *scbR*, maximum expression was reached only at late transition phase in LW33 compared to mid transition phase in LW34 (Figure 7 and Additional file 4AB).

### ScbR_M600_from *Streptomyces* shows reduced ability to bind to the *scbR* promoter sequence

**Figure 8:**
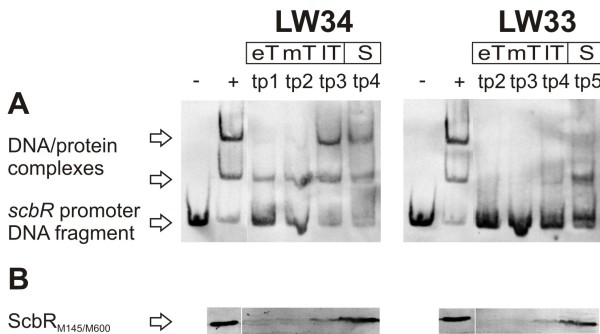
** Gel retardation assay and Western analysis using*S. coelicolor*cell-free extracts.** Cell-free extracts (CE) of *S. coelicolor* LW34 (*scbR*_M145_) and LW33 (*scbR*_M600_)obtained at four time points (tp 1–4) during different phases of growth indicated with eT, mT, lT and S (early, mid, late transition, and stationary phase; also see Table 2, GC 1) were used to determine presence and DNA binding ability of ScbR_M145/M600_. CE from *E. coli* JM101/pIJ6120 harbouring ScbR_M145_ was used as a positive control (indicated with a “+”). **A**. DNA binding abilities of ScbR_M145_ and ScbR_M600_ from *S. coelicolor* CE were tested using a DIG-labelled *scbR* promoter DNA fragment by gel retardation analysis. The *scbR* promoter DNA fragment and DNA/protein complexes are indicated by arrows. The DNA probe alone was used as a negative control (indicated with a “-“). **B**. Western analysis of ScbR. ScbR_M145_ (+, LW34) and ScbR_M600_ (LW33) was detected in similar amounts at corresponding time points in the two *S. coelicolor* strains. ScbR signals are indicated by an arrow.

Cell-free extracts (CE) of LW34 (*scbR*_M145_) and LW33 (*scbR*_M600_) were prepared from samples taken at different phases of growth (Table 2) corresponding to the sampling time points in the transcription analysis. DNA binding ability was determined in gel retardation assays using a digoxygenin-labelled *scbR* promoter fragment containing the ScbR binding site upstream of its own promoter [9] and freshly prepared protein extracts (Figure 8A; shown for GC 1). Equivalent amounts of total protein used for the gel retardation analysis were also analysed by Western hybridization using an antibody to ScbR to determine the relative abundance of ScbR in the extracts (Figure 8B). Extracts from LW34 from late transition and stationary phase completely shifted the operator DNA in the binding assay (Figure 8A). This is consistent with the appearance of ScbR as detected by Western blotting (Figure 8B). In contrast, all LW33 extracts failed to produce a full shift. Even in the presence of a higher amount of ScbR_M600_ compared to ScbR_M145_ (LW33 in stationary and LW34 in transition phase, respectively; Figure 8B) the observed shift of ScbR_M600_ was weaker, suggesting that the DNA binding ability of the mutant protein in LW33 is reduced. The same results were obtained with GC 2 (data not shown).

### *In vitro* analysis using freshly prepared protein expressed in *E. coli* indicates that the DNA and γ-butyrolactone binding abilities of ScbR_M145_ and ScbR_M600_ are comparable

**Figure 9:**
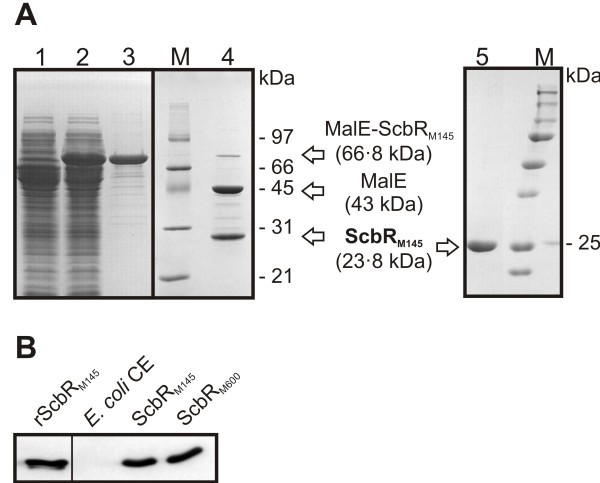
** Heterologous expression and Western analysis of ScbR. A**. Heterologous expression and purification of ScbR_M145_ for the creation of ScbR antibodies. Crude cell extracts from *E. coli* JM101 harbouring pTE88 before (lane 1) and after (lane 2) induction with 0.2 % (w/v) of L-rhamnose. The MalE-ScbR_M145_ fusion protein present in the induced fraction was then purified with an amylose resin (lane 3). MalE-ScbR_M145_ was cleaved with Factor Xa to separate MalE from ScbR_M145_ (lane 4). ScbR_M145_ was further purified with a heparin column (lane 5). Arrows show the protein bands representing each protein. Theoretical molecular weight of MalE-ScbR_M145_, MalE and ScbR_M145_ are noted in brackets. All protein fractions were analysed on 12 % (w/v) SDS-PAGE followed by staining with Coomassie blue. M denotes for prestained protein molecular weight ladders (SM0431 (old version) (Fermentas).and Precision Plus Protein “All Blue” Standard (BioRad)). **B.** Heterologous expression of ScbR_M145_ and ScbR_M600_ for gel retardation assays and Western analysis of both forms of ScbR. ScbR-antibodies were generated using recombinant ScbR_M145_ shown in (a). ScbR_M145_ and ScbR_M600_ were expressed in *E. coli* JM101/pIJ6120 and pTE58 harbouring *scbR*_M145_ and *scbR*_M600_, respectively. In Western analysis, ScbR was detected with a sample of the recombinant ScbR_M145_ (“rScbR_M145_”) and in cell-free extracts (CE) of *E. coli* JM101/pIJ6120 and pTE58 (“ScbR_M145_” and “ScbR_M600_”). In CE of *E. coli* JM101 harbouring the empty expression vector pIJ2925 (“*E. coli* CE”) no ScbR was found. Comparable amounts of ScbR were detected with same amounts of total CE proteins.

**Figure 10:**
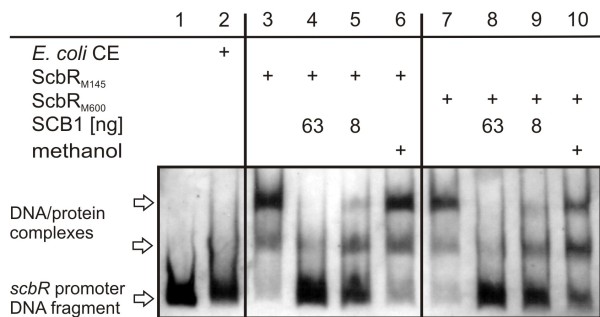
** Gel retardation assay using ScbR**_**M145**_**and ScbR**_**M600**_**from*E. coli* supplemented with SCB1.** DNA and γ-butyrolactone binding abilities of ScbR_M145_ and ScbR_M600_ from *E. coli* cell-free extracts (CE) were tested using a DIG-labelled *scbR* promoter DNA fragment and the *S. coelicolor* γy-butyrolactone SCB1 in a gel retardation assay. All samples contained the labelled DNA probe. Sample two contained CE of *E. coli*/pIJ2925 (“*E. coli* CE”), samples 3–6 and 7–10 of *E. coli* JM101/pIJ6120 and pTE58 (“ScbR_M145_” and “ScbR_M600_”). To samples 4, 5, 8 and 9 SCB1 dissolved in methanol was added in high (63 ng) and low (8 ng) amounts. Samples 6 and 10 were supplemented with same volumes of pure methanol. The *scbR* promoter DNA fragment and DNA/protein complexes formed with ScbR_M145/M600_ are indicated by arrows. DNA binding abilities of the two variants of ScbR were shown to be the same in the absence and the presence of the γ-butyrolactone.

**Figure 11:**
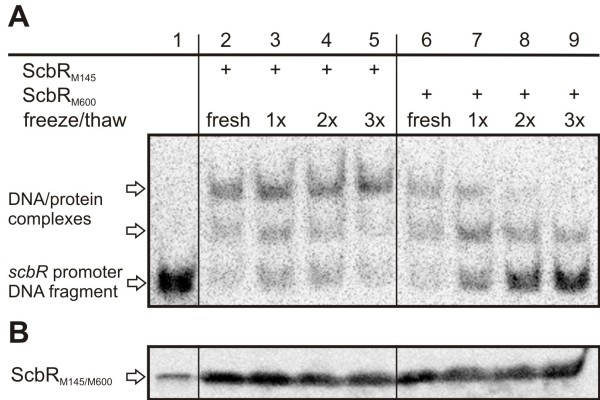
** Gel retardation assay and Western analysis using ScbR**_**M145**_**and ScbR**_**M600**_**from*E. coli* after freezing and thawing. A**. DNA binding abilities of ScbR_M145_ and ScbR_M600_ from *E. coli* cell-free extracts (CE) were tested using a DIG-labelled *scbR* promoter DNA fragment in a gel retardation assay. All samples contained the labelled DNA probe. Samples 2–5 and 6–9 contained CE of *E. coli* JM101/pIJ6120 and pTE58 (“ScbR_M145_” and “ScbR_M600_”), respectively, frozen and thawed up to three times before added to the DNA probe. The *scbR* promoter DNA fragment and DNA/protein complexes formed with ScbR_M145/M600_ are indicated by arrows. In contrast to ScbR_M145_, DNA binding of ScbR_M600_ was shown to be unstable under the conditions tested. **B**. Western analysis of ScbR. ScbR_M145_ was detected with a positive control sample of recombinant ScbR_M145_ (“rScbR_M145_”, lane 1) and in comparable amounts in all gel retardation assay samples harbouring ScbR_M145_ or ScbR_M600_ as described in A (lanes 2–9). ScbR bands are indicated by an arrow.

*In vivo* analysis in this study indicated that expression of mutant ScbR_M600_ in *S. coelicolor* leads to altered patterns of expression of the pathway-specific regulatory genes for Red (*redD*) and CPK (*cpkO*), the *scbA* and *scbR* genes known to be controlled by ScbR in response to altered butyrolactone concentrations, and to a decreased ScbR_M600_ DNA binding affinity. To investigate in more detail the effect that the point mutation may have on the ability to bind both butyrolactones and its cognate DNA operator sequence, ScbR_M145_ and ScbR_M600_ were overexpressed in *E. coli*. Freshly prepared *E. coli* CE containing equal amounts (Methods; Figure 9B) of the two forms of ScbR showed comparable DNA binding abilities in gel retardation analysis (Figure 10, lanes 3 and 7, and Figure 11A, lanes 2 and 6, respectively).

Addition of low (8 ng; Figure 10, lanes 5 and 9) and high (63 ng; Figure 10, lanes 4 and 8) amounts of the cognate γ-butyrolactone ligand SCB1 led to a partial and a complete loss of DNA binding, respectively, with both ScbR_M145_ and ScbR_M600_. Therefore, under the conditions tested, the γ-butyrolactone binding affinity of the two forms of ScbR expressed in *E. coli*were comparable.

### DNA binding ability of ScbR_M600_ expressed in *E. coli* is unstable

In gel retardation analysis using *E. coli* CE supplemented with 4 % (v/v) glycerol, DNA binding of the mutated ScbR_M600_ was reduced when stored at −20 °C for several days or weeks (data not shown). To further analyse this difference, fresh CE without glycerol was frozen and thawed up to three times at −20 °C and 37 °C, respectively, and the DNA binding affinity was tested by gel retardation analysis. To ensure equal amounts of protein for both ScbR_M145_ and ScbR_M600_, total protein concentrations of the CE were measured, and the abundance of ScbR present was confirmed by Western analysis (Figure 11B). Both proteins were active in freshly prepared and analysed CE (Figure 11A, lanes 2 and 6), but freezing and thawing of the proteins lead to a decrease (lane 7 and 8) and eventually to the loss (lane 9) of DNA binding with the mutant ScbR_M600_. Native ScbR_M145_ was not affected by the treatment (lanes 3 to 5). Western analysis indicated that freezing and thawing had no effect on the amount of protein detected (Figure 11B) indicating that the loss of DNA binding ability was not due to protein degradation. This suggests that compared to ScbR_M145_, the DNA binding ability of ScbR_M600_ is significantly less stable. This is also supported by the decrease in DNA binding affinity observed with the mutant protein, but not with ScbR_M145_, in the presence of methanol (Figure 10, lanes 6 and 10). This instability of ScbR_M600_ DNA binding activity was also seen from crude extracts obtained from *Streptomyces* (data not shown).

## Discussion

### A variant form of the γ-butyrolactone receptor ScbR in *S. coelicolor*

In addition to ScbR from the sequenced strain M145 (ScbR_M145_), a natural variant of the protein, ScbR/R120S (ScbR_M600_), was found in strain M600 (this study). The underlying point mutation in the *scbR* gene, c358a, was also found in strain A3(2) N2, another *S. coelicolor* A3(2) derivative independent from both M145 and M600 [4]. All 14 further strains tested showed the M145 genotype, including *S. coelicolor* W3443, the proposed progenitor of all strains described [4], and *scbR*_M600_ is thus regarded as a mutant variant. When mapping the known instances of this variant onto the lineage of *S. coelicolor* described by Kao and co-workers [4], our results indicate that the point mutation c358a occurred on two independent occasions, in strains M600 and A3(2) N2, perhaps indicating a selective pressure or an evolutionary relevance for this change under certain conditions.

### The amino acid change in ScbR_M600_ impairs DNA binding in *S. coelicolor*

Compared to ScbR_M145_, DNA binding was reduced with ScbR_M600_ (from *S. coelicolor* LW34 and LW33, respectively; Figure 8). This difference is surprising since the functional localization of the altered amino acid 120 does not suggest an effect of the mutation in *scbR*_M600_ on the DNA binding domain of the protein. Furthermore, Horinouchi and co-workers described the helix-turn-helix motifs of mutant ArpA/W119A as still able to bind DNA [22]. However, in the ArpA study the intensity of the shifted band observed in the absence of A-factor was noticeably weaker for the mutated ArpA/W119A than for the wild type ArpA [22]. This also implies a reduced DNA binding affinity of ArpA/W119A, and thus suggests an effect of the amino acid change W119A in ArpA similar to that observed with ScbR_M600_ in this study. It is interesting to note that both variants of ScbR expressed in *E.coli* did not have any difference in DNA binding activity. This may suggest another component in *S. coelicolor* is responsible for the loss of the DNA binding activity of ScbR.

### The mutation in ScbR_M600_ has no detectable effect on γ-butyrolactone binding

The crystal structure of a ScbR paralogue in *S. coelicolor*, CprB, is regarded as representative for γ-butyrolactone receptors in streptomycetes. Functional domains for dimerization, DNA binding by helix-turn-helix motifs, and γ-butyrolactone binding have been proposed. DNA binding of CprB was shown experimentally [22], but a putative cognate γ-butyrolactone ligand has yet to be identified [14]. From the CprB amino acid sequence, amino acid 120 in ScbR is proposed to form part of a central α-helix (α7) in the regulatory region (Figure 3). The adjacent tryptophan residue W121 is predicted to be directly involved in forming the γ-butyrolactone binding pocket and is highly conserved among *Streptomyces* γ-butyrolactone receptors [14]. Mutation of the corresponding W119 in the A-factor receptor protein ArpA, a ScbR homologue in *S. griseus*, abolished γ-butyrolactone binding [22]. It therefore seems reasonable to suggest that a change in the amino acid residue 120 adjacent to the crucial W121 in ScbR, from a large (174 Da) basic arginine to a small (105 Da) neutral serine, could affect ligand binding affinity.

However, no difference in ligand binding was observed between ScbR_M145_ and ScbR_M600_ (Figure 10), and it is interesting to note that ScbR amino acid 120 is not conserved among γ-butyrolactone receptor proteins. The corresponding residues in other ScbR homologues consist of members of all classes of amino acids and of hugely differing sizes (e.g., aspartate (acidic, 133 Da) in ArpA (*S. griseus*); arginine (basic, 174 Da) in ScbR_M145_; asparagine (hydrophilic, 132 Da) in FarA (*S. lavendulae*); alanine (hydrophobic, 89 Da) in SpbR (*S. pristinaespiralis*)), all adjacent to the conserved tryptophan residue [14]. The high level of variability at this position suggests residue 120 does not in fact play a significant role in γ-butyrolactone binding.

### ScbR_M600_ leads to altered gene expression and has some affects on γ-butyrolactone production

Expression of *scbA* is delayed in LW33 (Figure 7). This could be a direct effect of the impaired DNA binding ability of ScbR_M600_ since ScbR was previously shown to be necessary for *scbA* expression [9]. Consistently, late expression of the γ-butyrolactone synthase gene coincides with a slight delay in the production of the small signalling molecules in LW33 (Figure 6).

Also *scbR* expression is somewhat delayed at similar transcription levels in the presence of the M600-type protein, but not as clearly as with *scbA* (Figure 7). This is at first glance surprising, since a reduction in DNA binding of the auto-repressor might be expected to result in a higher or early expression of *scbR*_M600_. However, also in Western analysis no increase in the amount of ScbR_M600_ in LW33 was observed (Figure 8B). γ-butyrolactones abolish the DNA binding of ScbR and are active in nM concentrations (Figure 10) [9]. The small reduction in γ-butyrolactone production observed in LW33 (Figure 6) may promote binding of ScbR_M600_ to its target sites *in vivo*, counteracting the impaired binding ability of the mutant protein.

In LW33, expression of the pathway-specific regulator genes for the *red* and the *cpk* antibiotic biosynthesis gene clusters was delayed compared to that in LW34 (Figure 7 and Additional file 4AB). However expression of *scbRscbAredDcpkO* and *actII*-orf4 has been shown to be highly similar in M145 and M600 in an extensive genome-wide study by Weaver and co-workers [4]. Despite these differences, antibiotic production was similar in the LW33 and LW34. Regulation of production of these antibiotics is complex and known to be affected by many additional factors [23].The *cpk* gene cluster, for example, was shown to be also regulated by the RapA1/A2 two-component system [24] and the global regulator DasR [25]. It appears that the changes in expression of the regulatory genes observed here are over-ridden by other control mechanisms and so do not result in any observable change in the antibiotic phenotype. Furthermore, production of other, known and unknown, secondary metabolites in *S. coelicolor* might impair the synthesis of Act, Red and yCPK, e.g. by competition for common precursors from primary metabolism. Further experiments will be needed to reveal the detailed effects of the mutation in ScbR_M600_ on the delicately balanced butanolide system in *S. coelicolor*.

Despite the somewhat impaired DNA binding ability of the mutant ScbR_M600_, our result show that strain LW33 clearly differs from a M145Δ*scbR* in-frame deletion mutant (M752). In comparison to M145, the latter strain is delayed in Red production, transcribes *scbR* early, and expresses *cpkO* early and constitutively [9,10]. Evidently, the single R120S amino acid change has a different impact on the *S. coelicolor* butanolide system and related secondary metabolites when compared to a complete deletion. The antibiotic production in M600 also resembles that of the M145Δ*scbR* in-frame deletion mutant (M752) where all antibiotics are produced later than M145. We have shown that the cause of this antibiotic production phenotype is not due to the point mutation in ScbR alone.

### Structural effect of amino acid change R120S on the DNA binding domain of ScbR_M600_

DNA binding of ScbR_M600_ was impaired in both the natural producer *S. coelicolor* and, after harsh low-temperature treatment, in *E. coli* CE. The decreased binding ability is therefore most probably not related to specific properties of the *S. coelicolor* cellular background, e.g. a hypothetical “deactivation” mechanism of the transcriptional regulator ScbR, and it is more likely that the amino acid change is causing a structural change affecting the DNA binding domain. This is also supported by the fact that a loss of ScbR_M600_ DNA binding could be induced by freezing and thawing of the protein (Figure 11A), and the observation that this loss was prevented by the addition of 43 % (v/v) of glycerol (data not shown). However, modelling of both ScbR variants based on the crystal structure of the ScbR paralogue CprB [14] does not reveal an obvious destabilizing effect of the mutation R120S in ScbR_M600_ (data not shown; Dirk Linke, personal communication). Also, protein aggregation, specifically of the mutated ScbR_M600_, could cause these effects, whereas Western analysis data shown in Figure 8B and Figure 11B clearly exclude degradation of the protein as an explanation for our observations. Prediction of enzymatic cleavage using the ExPASy Peptide Cutter (Swiss Institute of Bioinformatics (SIB)) (http://expasy.org/tools/peptidecutter/) only revealed an additional cleavage site for pepsin at pH1.3 (and not pH > 2) in ScbRM600, and the loss of one site each for Arg-C proteinase, clostripain and trypsin (data not shown).

### The mutation in ScbR_M600_ is not responsible for the absence of the protein in vegetative mycelium of strain M600

To our surprise and in contrast to M145, ScbR_M600_ was absent during vegetative growth in M600 as shown by 2D gel (Figure 1) and Western analysis (data not shown). However, ScbR_M600_ was present in vegetative mycelium of *S. coelicolor* LW33 harbouring* scbR*_M600_ in an M145 genetic background. The amounts were comparable to ScbR_M145_ in the control strain LW34 (Figure 8B) and in M145 (data not shown). This suggests that it is the M600 genetic background, and not the amino acid change in ScbR_M600,_ that is the reason for the absence of ScbR during vegetative growth of M600. Whether the altered properties of ScbR_M600_ are an adaptation to its synthesis in the spores of *S. coelicolor* M600 and/or to a putative modified function of the regulator which again might indicate evolutionary relevance remains to be determined. Detailed knowledge of the butanolide regulatory system in *S. coelicolor* might allow rational construction of strains with improved timing and levels of antibiotic production.

## Conclusions

Among at least five paralogues in *S. coelicolor* (CprA, CprB, SCO6323, ScbR and ScbR2), ScbR is the only γ-butyrolactone receptor experimentally shown to bind a cognate signalling molecule. Recently, SCO0608 (SlbR) was shown to also bind to butyrolactones with less specificity compared to ScbR, *in vitro* and shares similar recognition sites as ScbR. However the effect on antibiotic production is much less dramatic than that of ScbR [19,21]. Therefore ScbR still plays a key role in the *S. coelicolor* butanolide system involved in the regulation of the antibiotics Act and Red, and of the *cpk* secondary metabolite gene cluster (reviewed in [5]). We identified a variant ScbR with only one amino acid exchange in *S. coelicolor* M600 that is impaired in its DNA binding abilityandalters the expression of the pathway-specific regulators of two antibiotic biosynthetic gene clusters. This demonstrates that the γ-butyrolactone receptor, ScbR, plays a key role in the SCB regulatory cascade and in determining the onset of the expression of the antibiotic regulatory genes.This variant was only found in two *Streptomyces coelicolor* strainswhich most likely arose in laboratory conditions. To understand how this variant was selected twice may shed light on the evolutionary diversity of signalling receptor molecules.

### Availability of supporting data

The data sets supporting the results of this article are included within the article and its additional files.

## Methods

### Bacterial strains, plasmids and cosmids

Strains and vectors used in this study are listed in Table 1 and Additional file 5. *Streptomyces* was manipulated as described previously [18]. *Escherichia coli* was grown and transformed according to [16].

### Culture conditions

For culturing *E. coli*, liquid LB [16] or LB agar supplemented with appropriate antibiotics was used. For genomic DNA isolation *Streptomyces* was grown in liquid YEME/TSB (1:1) medium as described [18]. MS medium [18] was used to harvest spores according to [18]. To determine the viable spore concentration, dilution series of spore suspensions were plated on MS or DNA medium (DNagar) [18] and the number of colony forming units was determined. DNAgar supplemented with 325 mM (final conc.) L-Glutamic acid monosodium salt (Glu-DNAgar) [12] was used for determination of secondary metabolite production. For liquid *Streptomyces* cultures, strains were cultivated using SMM as previously described [9, 19]. Interspecific conjugation was done as described previously [9].

### PCR and DNA sequencing

Amplification of DNA by PCR [26] was done with Taq polymerase (Fermentas), ProofStart polymerase (Qiagen) or the Expand High Fidelity DNA System (Roche). DNA sequence analysis was carried out by Sequence Laboratories, Göttingen, Germany.

### DNA manipulation, plasmid transformation and intergeneric transfer

Plasmid DNA isolation, restrictions and cloning experiments were carried out as described in [16]. *Streptomyces* was manipulated as described in [2], genomic DNA was isolated according to [27].

### 2D gel electrophoresis and MALDI-TOF mass spectrometry

Protein extracts were prepared from typically 25 ml culture samples according to [28] and mycelial pellets were stored at -80 °C until use.To prepare total protein extracts from spores, spores harvested from cultures grown on MS plates [18] and stored frozen in glycerol at −80 °C were thawed on ice, washed once with ice cold wash buffer [28], then transferred to a mortar submerged in liquid nitrogen together with an equal volume of washed glass beads (Sigma G-8893, 106 microns). The spores were then ground thoroughly under liquid nitrogen until an even colour and fine consistency (about 5 min), and the resulting frozen powder stored at −80 °C until use. Frozen aliquots were suspended in spore protein buffer (50 mM DTT, 4 mM Pefabloc SC protease inhibitor, 40 mM Tris pH 9.0, 1 mM EDTA, 1 mM EGTA and 2 % (w/v) SDS), sonicated briefly (Sanyo Soniprep 150; 2 x 5 second bursts at amplitude 7.5 microns), and then boiled for 10 min. After cooling, cell debris and glass beads were removed, the protein extract was cleaned up, and protein pellets were finally dissolved and stored frozen in aliquots at −80 °C until use as described in [28]. Protein extracts from mycelia and spores were subjected to 2D gel electrophoresis as detailed in [28]. The strip used for the separation was an 18 cm pH 5.5-6.7 IPG strip (Amersham Biosciences). Gels were stained with Sypro Ruby (Bio Rad) according to the manufacturer’s instructions, and scanned using the Perkin-Elmer ProXPRESS proteomic imaging system using excitation and emission wavelengths of 480 nm and 630 nm, respectively.

Protein spots of interest were excised from stained gels using the Investigator ProPic robot from Genomic Solutions, and identified by tryptic or chymotryptic digestion and MALDI-TOF mass spectrometry using a Micromass Q-TOF 2 mass spectrometer as previously described [28]. Identification of proteins from peptide mass fingerprint data was performed according to [28].

### Overexpression and purification of ScbR_M145_ for the generation of ScbR antibodies

The *scbR*_M145_ coding sequence was amplified by PCR from the cosmid SCAH10 [29] using primers MalE-ScbR1 and MalE-ScbR2 (Additional file 2). The PCR product was gel-purified and ligated to pDRIVE (Qiagen), yielding pDRIVE-ScbR. This plasmid was digested with *Bam*HI and *Hind*III and the fragment corresponding to *scbR*_M145_ was ligated to the digested *Bam*HI/*Hind*III vector pTST101 [30] to allow the translational fusion of *scbR*_M145_ with *malE*, leading to pTE88. The sequence of *scbR*_M145_ and the translational fusion were confirmed by DNA sequencing.

*E. coli* JM101 was transformed with pTE88. LB with 50 μg ampicillin ml^-1^ was inoculated to 1/100 volume with the overnight pre-culture and cells were grown at 37 °C until OD_600_ reached 0.5. L-rhamnose (0.2 % (w/v) final concentration) was added for induction. After a further 2 h of incubation the induced cells were harvested and washed twice with chilled Column Buffer (CB) (20 mMTris-HCl, 200 mM NaCl, 1 mM EDTA), resuspended in chilled CB and disrupted using a French-Press. The soluble fraction containing MalE-ScbR_M145_ was aliquoted and frozen at -70 °C until use. The MalE-ScbR_M145_ was further purified by affinity chromatography with amylose resin (New England Biolabs) using a Bio-Logic FPLC system (Bio-Rad). After elution with CB supplemented with 10 mM maltose, positive fractions (0.5-5 mg ml^-1^) detected by UV absorbance at 280 nm and shown to contain MalE-ScbR_M145_ (66.4 kDa) by SDS-PAGE were pooled. ScbR_M145_ was cleaved from MalE by the specific protease Factor Xa (10 μg ml^-1^ final concentration, New England Biolabs). Complete cleavage was determined by SDS-PAGE and cleavage was stopped by adding 2x Protease Inhibitor Cocktail (Roche). ScbR_M145_ (23.8 kDa) was further purified to homogeneity by affinity chromatography using a heparin column (Amersham Biosciences) coupled to the Bio-Logic FPLC system (Bio-Rad). ScbR_M145_ was eluted using a continuous salt gradient (0.2-2 M NaCl in CB). Positive fractions were checked on SDS-PAGE for presence and purity of ScbR_M145_ (Figure 9). The purified protein (0.8 mg) was used to generate antibodies in rabbits (Eurogentec S.A., Belgium).

### SDS-PAGE and Western analysis

Cell-free extracts (CE) or purified ScbR were resolved by SDS-PAGE (12 % (w/v) resolving SDS-polyacrylamide gels) according to Laemmli’s procedure [31]. Following electrophoresis, resolved bands were visualized by Coomassie brilliant blue staining. For Western analysis, proteins separated on SDS-PAGE gels were transferred to a nitrocellulose membrane by immersion or semi-dry blotting. Immunodetection of ScbR was carried out by using rabbit antiserum raised against ScbR_M145_ (this study) and horseradish peroxidase-conjugated goat anti-rabbit IgG (Bio-Rad) as a secondary antibody with Roche’s CSP-*Star* (Figure 11B and Figure 9B) or NBT/BCIP (Figure 7B) as a substrate. In the former case, Super RX Medical X-ray Film (NIF100) (Fuji film) and a Lumi-Imager F1 (Roche) (Figure 11B) or a Konica QX-150U Medical Film Processor (Figure 9B) were used for detection.

### Construction of the ScbR_M600_ expression plasmid and expression of both forms of ScbR in *E. coli*

A *scbR*_M600_ expression vector, pTE58, was constructed as described in detail in Additional file 6A, B. Plasmid pTE58 and the *scbR*_M145_ expression construct pIJ6120 [9] contain the two forms of *scbR* with its own promoter region cloned behind the *lacZ* promoter in a pIJ2925 backbone. Expression constructs pTE58 and pIJ6120 were partially sequenced and differ only by the natural point mutation in *scbR*_M600_ (this study). *E. coli* JM101 was transformed with pTE58 and pIJ6120 for heterologous expression of ScbR_M600_ and ScbR_M145_ and CE was used for Western hybridisation analysis and gel retardation assays.

### Cell-free extract preparation

For *E. coli* cell-free extract (CE), 10 ml LB overnight cultures of *E. coli* JM101 harbouring pIJ2925, pIJ6120 and pTE58 were inoculated at a 1/100 concentration in 50 ml LB without glucose. Cultures were incubated at 37 °C for 2.5 h or until OD_600_ 0.7-0.8 and induced with 1 mM (final concentration) IPTG. After 3 h of further incubation cells were harvested and washed twice with ice cold disruption buffer (50 mM TrisHCl pH 7.9, 1 mM EDTA pH 8.0, 1 mM DTT, 1x (final conc.) complete EDTA-free protease inhibitor (Roche)) before being resuspended in 400 μl disruption buffer. For the *E. coli* CE used as positive control in Figure 7 the disruption buffer contained 20% (v/v; final conc.) glycerol. Cells of 100 μl aliquots were collected by centrifugation, the supernatant was removed completely and the cell pellet was frozen at −80 °C. To prepare CE, cells were resuspended in 150 or 200 μl disruption buffer and were disrupted by sonication. The cell lysate was clarified by centrifugation. Total protein concentration of the supernatant was determined using the BCA Protein Assay kit (Pierce) (for Figure 10 and Figure 9B) or a NanoDrop spectrophotometer (Thermo Fisher Scientific) (for Figure 7 and Figure 11). The freshly prepared CE was used immediately (Figure 10 and Figure 9B (100 μg total CE protein used), and Figure 7 (50 μg)). For Figure 11, the CE (130 μg total protein) was frozen and thawed up to three times at -20 °C (15 min) and 37 °C (5 min) before being applied to gel retardation assays and Western hybridisation analysis.

For *S. coelicolor* CE, cells from 25 ml samples of individual 60 ml SMM liquid cultures were collected at different phases of growth (Table 2, GC 1). Fresh CE was prepared with all cells obtained as described for *E. coli* using 100, 150 or 200 μl disruption buffer with glycerol. 500 μg total CE protein was used for Western analysis and gel retardation assays shown in Figure 8.

### Gel retardation analysis

Gel retardation experiments were carried out as described previously [9] using the Roche DIG Gel Shift Kit (Roche cat No. 1635352). From a genomic DNA isolate of *S. coelicolor* M145, a 177 bp PCR fragment containing the ScbR binding site in the promoter region of *scbR*[9] was amplified using primers ETS6 and ETS10 and then was DIG-labelled according to the manufacturer’s manual. For each sample approximately 0.23 ng (Figure 11A) and 1.8 ng (Figure 10) were used. For the results shown in Figure 8A, primer ETS10 was replaced by ETS10_DIG(5’) with digoxygenin linked to the 5’-end of the primer to obtain a labelled probe directly from PCR of which 5 ng was used in each sample (all primers are listed in Additional file 2). In some cases, 8 or 63 ng of chemically synthesised *S. coelicolor* γy-butyrolactone SCB1 was added to the mixture prior to incubation (Figure 10). For Figure 10, a Super RX Medical X-ray Film (NIF100) (Fuji film) and a Konica QX-150U Medical Film Processor were used for detection. Pictures for Figure 8A and Figure 11A were obtained with the Lumi-Imager F1 (Roche) and the Luminescent Image analyzer LAS-4000 (Fuji film), respectively.

### Complementation of a *S. coelicolor* M145Δ*scbR* mutant (M752) with ScbR_M145_ and ScbR_M600_ by chromosomal replacement

*scbR*_M145_and *scbR*_M600_ complementation vectors were constructed as described in detail in Additional file 7A, B, C, D, E. Resulting plasmids pTE212 and pTE214 contain a 2.4 kb fragment of *scbR*_M600_ and *scbR*_M145_ and its flanking regions, respectively, in the conjugative, non-integrative vector pKC1132 [32] which is non-replicating in *S. coelicolor*. The 2.4 kb inserts were sequenced and differ only by the natural point mutation in *scbR*_M600_ (this study) and an additional silent point mutation, c636t, in *scbR*_M145_ of pTE214 with no effect on the amino acid sequence of ScbR_M145_. pTE212 and pTE214 were transferred into *S. coelicolor* M752 by conjugation via *E. coli* ET12567/pUZ8002. Single- and double-crossover mutants were selected as described in [9], but using DNAgar for non-selective growth, yielding strains LW33 and LW34 in which the truncated *scbR*_M145_ region of the M145Δ*scbR*_M145_ mutant M752 was replaced by *scbR*_M600_ and *scbR*_M145_, respectively, in a M145 chromosomal background. Presence of full-length *scbR* genes at the right chromosomal location was confirmed by PCR using primers ETseq3 and ETS7 and Southern analysis [18] using a PCR-generated 484 bp probe (primers scbArt1 and scbArt2 labelled with a DIG DNA labelling kit (Roche) binding to a 2.37 kb DNA fragment of *Nco*I digested chromosomal DNA in the presence of full-length *scbR* (Figure 4A,B). With the in-frame deletion in *scbR*_M145_ in M752, an *Nco*I restriction site disappears and the probe binds to a 3.2 kb DNA fragment. The two variants of *scbR* in LW33 and LW34 were confirmed by specific PCR using primers RCseq31 and scbR-M145_c358 or scbR-M600_c358a (Figure 4C) and by DNA sequence analysis of the full-length *scbR* PCR product shown in Figure 4C (data not shown). All primers are listed in Additional file 2.

### Reverse transcription and quantitative RT-PCR

RNA was isolated as previously described [10] from LW33 and LW34 grown in duplicate in liquid SMM at different times of growth. cDNA synthesis and quantitative RT-PCR was conducted as reported in [12].Three technical replicates per gene and time point were done and *hrdB* was used as endogenous control. All primers used for the qRT experiments are listed in Additional file 2. The averaged data is shown in Additional file 4A and the original study data are available as Additional file 8, data for growth curve 2 are shown in Figure 7.

### Detection of γ-butyrolactone production in liquid *S. coelicolor* cultures

*S. coelicolor* LW33 and LW34 were grown in liquid SMM at 30 °C. At four time points (Table 2, GC 2), γ-butyrolactones were extracted from the culture supernatant as described in [9] and detected by the kanamycin bioassay [21].10^9^ spores per plate of the indicator strain were plated out for confluent lawns on DNAgar plates containing 5 μg kanamycin ml^-1^. Each 5 μl of the γ-butyrolactones extracts, as well as 125 ng of the γ-butyrolactone SCB1 as positive control and the solvent methanol as negative control were spotted in the middle of the plates. After incubation for 3 days at 30 °C growth of the indictor strain was determined; pictures were taken from the bottom (Figure 6).

### Determination of secondary metabolite production

4 x 10^*7*^ spores per plate of *S. coelicolor* were streaked out for a 2.5 x 2.5 cm square on Glu-DNAgar, and pictures were taken from the bottom at different phases of growth to determine production of pigmented secondary metabolites (Figure 5). Antibiotic production in liquid cultures was determined as described previously [33].

## Competing interests

None of the authors have a competing interest.

## Authors’ contributions

MG, AH, RB, and PP conducted the experiments. AH and ET designed the experiments. MG, AH and ET analysed the results and wrote the manuscript. All authors read and approved the final manuscript.

## Supplementary Material

Additional file 1 Data from the chymotryptic digests. Click here for file

Additional file 2 Primers used for (qRT-)PCR experiments. Click here for file

Additional file 3 **Transcriptional analysis of *redD*,*actII*-orf4,*cpkO*,*scbA*and*scbR***_**M145/M600**_**using qRT-PCR in GC 1.A.** qRT-PCR analysis of the transcription of *redD*, *actII*-orf4, *cpkO*, *scbA* and *scbR*_M145/M600_ using cDNA synthesized from RNA isolated from liquid SMM cultures of *S. coelicolor* LW34 (*scbR*_M145_) and LW33 (*scbR*_M600_). Samples were taken at four time points (tp 1–4) during different phases of growth indicated with eT, mT, lT and S (early, mid, late transition, and stationary phase). Gene expression is shown as fold-change relative to the LW34 time point 1 early transition phase sample. Error bars indicate the standard deviation (see data in Additional file 3, GC 1). **B**. Numerical data from of the original qRT-PCR results. Click here for file

Additional file 4 **Transcriptional analysis of *****redD*****,*****actII*****-orf4,*****cpkO*****,*****scbAandscbR***_**M145/M600**_**using qRT-PCR in GC 1.A.** qRT-PCR analysis of the transcription of *redD*, *actII*-orf4, *cpkO*, *scbA* and *scbR*_M145/M600_ using cDNA synthesized from RNA isolated from liquid SMM cultures of *S. coelicolor* LW34 (*scbR*_M145_) and LW33 (*scbR*_M600_). Samples were taken at four time points (tp 1–4) during different phases of growth indicated with eT, mT, lT and S (early, mid, late transition, and stationary phase). Gene expression is shown as fold-change relative to the LW34 time point 1 early transition phase sample. Error bars indicate the standard deviation (see data in Additional file 3, GC 1). **B**. Numerical data from of the original qRT-PCR results. Click here for file

Additional file 5 Plasmids and cosmids used in this study. Click here for file

Additional file 6 **Construction of the ScbR**_**M600**_**expression vector pTE58. From a genomic DNA isolate of *S. coelicolor*strain M600, a 931 bp PCR fragment containing the*scbR***_***M600***_**coding sequence and the*scbR*promoter region was amplified using primers ETS3 and ScbR2 (Additional file **2**).** The PCR product was gel-purified and ligated to pDRIVE (Qiagen), yielding pTE51 of which the insert contains not only the natural mutation c358a in *scbR*_M600_, but also additional mutations: a30g in *scbA* is located in the promoter region of *scbR*, and nucleotide change t587a in the coding sequence of *scbR*_*M600*_, respectively. The point mutation t587a leads to an amino acid change, D196V, in ScbR_M600_ (data not shown). A 948 bp pTE51/*Eco*RI *scbR*_*M600*_fragment was cloned into the pUC18 derivate pIJ2925 to gain pTE53 containing all three described mutations (**A**). A 844 bp pTE53/*Pst*I fragment containing the *scbR* promoter region and nucleotides 1-535 of the *scbR*_*M600*_coding region was ligated into a 3103 bp pIJ6120/*Pst*I vector fragment [9] containing nucleotides 536–648 of the *scbR*_*M145*_coding region, yielding pTE56. Partial sequence analysis of pTE56 revealed only the *scbR* promoter mutation a30g in *scbA* and the expected coding sequence mutation c358a of *scbR*_*M600*_. A 851 bp pTE56/*Sac*II fragment containing nucleotides 54–304 of *scbR*_*M600*_was cloned into a 3047 bp pIJ6120/*Sac*II vector fragment containing the *scbR* promoter region and nucleotides 1–53 and 305–648 of the *scbR*_*M145*_coding sequence, yielding pTE58 with *scbR*_* M600*_ and the *scbR* promoter region without additional undesired mutations in the same orientation as the IPTG-inducible *E. colilacZ* promoter (**B**). The *scbR*_*M145*_ expression construct pIJ6120 [9] and pTE58 differ only by the natural mutation, c358a, in *scbR*_*M600*_ and were used for the heterologous expression of ScbR_M145_ and ScbR_M600_ in *E. coli*. Linear DNA fragments are named and a scale is given with indicated base pair (bp) units. Plasmids are named and represented by black circles. Genes are indicated by dark arrows and labelled with gene names. For incomplete genes the missing part is indicated by an apostrophe at the beginning or the end of the gene name. Antibiotic resistance genes are abbreviated with “*amp*” for ampicillin and “*apra*” for apramycin. “*lacZ’*” denotes for the LacZ α-peptide coding sequence. The position of the described mutations is indicated by labelled grey boxes on the scales and in the plasmid maps; relevant restriction sites are shown with enzyme names. Thick black lines with arrows indicate ligation events; big black arrows with enzyme names indicate corresponding restriction steps. Click here for file

Additional file 7 **Construction of the complementation vectors pTE212 and pTE214.** From genomic DNA isolates of *S. coelicolor* strains M145 and M600, a 2406 bp PCR fragment containing the *scbR*_M145/M600_ coding sequence and flanking regions was amplified using primers BamRCseq31enh and BamETseq1 (Additional file 2). PCR products were gel-purified and ligated to pGEM-T EASY (Promega), yielding pTE63 harbouring *scbR*_M145_ (**A**), and pTE64 (**B**) and pTE203 (**C**) harbouring *scbR*_M600_. The inserts of the plasmids were sequenced and pTE63 was found to contain mutations in *scbR*_M145_ (silent mutation c636t) and *scbA* (c308t leading to A103V). pTE64 contains a mutation in *scbA* (t77c leading to M26T), and pTE203 in *scbB* (c644t leading to A215V). Two 1405 bp pTE203/*Pst*I fragments were introduced by tandem integration into a 4016 bp pTE64/*Pst*I vector fragment to give pTE211, which is pGEM-T EASY with a 2394 bp *Bam*HI *scbR*_*M600*_fragment without any additional mutations and an additional 1.4 kb *Bam*HI fragment. The 2394 bp pTE211/*Bam*HI fragment was cloned into pKC1132 to give pTE212 (**D**). A 1269 bp pTE63/*Nco*I fragment containing the silent mutation c636t in *scbR*_M145_ was cloned into a 4152 bp pTE211/*Nco*I vector fragment to give pTE213, which is pGEM-T EASY with a 2394 bp *Bam*HI *scbR*_*M145*_fragment with only the silent mutation. The 2394 bp pTE213/*Bam*HI fragment was cloned into pKC1132 to give pTE214 (**E**). Linear DNA fragments are named and a scale is given with indicated base pair (bp) units. Plasmids are named and represented by black circles. Genes are indicated by dark arrows and labelled with gene names. For incomplete genes the missing part is indicated by an apostrophe at the beginning or the end of the gene name. The ampicillin antibiotic resistance gene is abbreviated with “*amp*”. The position of the described mutations is indicated by labelled grey boxes on the scales and in the plasmid maps; relevant restriction sites are shown with enzyme names. Thick black lines with arrows indicate ligation events; big black arrows with enzyme names indicate corresponding restriction steps. Click here for file

Additional file 8 Excel file containing original qRT-PCR results. Click here for file
