# Supplementary material for: Characterisation of a natural variant of the γ-butyrolactone signalling receptor
Source: BMC Res Notes. 2012 Jul 27;5:379. doi: 10.1186/1756-0500-5-379 (PMC3461410; doi:10.1186/1756-0500-5-379)
Supplement: Additional file 1 — Data from the chymotryptic digests. [file 1756-0500-5-379-S1.pdf]

Additional file 1D

Additional MALDI-TOF data

Unmodified M145 ScbR (chymotrypsin digest):

75 ppm mass accuracy; 22 matched peptides; 87% coverage; MOWSE score = 100

1 MAKQDRAIRT RQTILDAAAQ VFEKQGYQAA TITEILKVAG VTKGALYFHF
51 QSKEELALGV FDAQEPPQAV PEQPLRLQEL IDMGMLFCHR LRTNVVARAG
101 VRLSMDQQAH GLDRRGPFRR WHETLLKLLN QAKENGELLP HVVTTDSADL
151 YVGTFAGIQV VSQTVSDYQD LEHRYALLQK HILPAIAVPS VLAALDLSEE
201 RGARLAAELA PTGKD

Start	End	Observed	Mr(expt)	Mr(calc)	Delta	Miss	Sequence
23	47	2652.5436	2651.5363	2651.4533	0.0830	3	F.EKQGYQAATITEILKVAGVTKGALY.F
28	47	2047.2069	2046.1997	2046.1724	0.0273	2	Y.QAATITEILKVAGVTKGALY.F
48	61	1651.9032	1650.8959	1650.8405	0.0554	4	Y.FHFQSKEELALGVF.D
48	75	3151.6526	3150.6453	3150.5661	0.0792	5	Y.FHFQSKEELALGVFDAQEPPQAVPEQPL.R
51	58	917.4995	916.4922	916.4865	0.0057	1	F.QSKEELAL.G
76	87	1497.7870	1496.7798	1496.7367	0.0431	3	L.RLQELIDMGMLF.C 2-Oxidation (M)
92	103	1311.8345	1310.8272	1310.7894	0.0378	0	L.RTNVVARAGVRL.S
104	118	1730.8395	1729.8322	1729.7954	0.0368	1	L.SMDQQAHGLDRRGPF.R Oxidation (HW)
113	118	747.4277	746.4204	746.3823	0.0381	0	L.DRRGPF.R
119	126	1110.6295	1109.6223	1109.6094	0.0129	2	F.RRWHETLL.K
119	126	1126.6293	1125.6220	1125.6043	0.0177	2	F.RRWHETLL.K Oxidation (HW)
119	126	1142.6222	1141.6149	1141.5992	0.0157	2	F.RRWHETLL.K 2-Oxidation (HW)
127	150	2605.4042	2604.3969	2604.3758	0.0211	3	L.KLLNQAKENGELLPHVVTTDSADL.Y
127	151	2768.5430	2767.5357	2767.4391	0.0965	4	L.KLLNQAKENGELLPHVVTTDSADLY.V
130	151	2414.2440	2413.2367	2413.1761	0.0607	2	L.NQAKENGELLPHVVTTDSADLY.V
152	175	2712.3910	2711.3837	2711.3190	0.0647	3	Y.VGTFAGIQVVSQTVSDYQDLEHRY.A
156	175	2308.1621	2307.1548	2307.1131	0.0417	2	F.AGIQVVSQTVSDYQDLEHRY.A
176	192	1783.1573	1782.1500	1782.1131	0.0369	2	Y.ALLQKHILPAIAVPSVL.A
179	192	1485.9502	1484.9429	1484.9078	0.0351	0	L.QKHILPAIAVPSVL.A
193	205	1400.7960	1399.7887	1399.7419	0.0468	2	L.AALDLSEERGARL.A
193	215	2354.3136	2353.3063	2353.2237	0.0826	4	L.AALDLSEERGARLAAELAPTGKD.
196	215	2099.1165	2098.1093	2098.0654	0.0439	3	L.DLSEERGARLAAELAPTGKD.

M600 ScbR (chymotrypsin digest):

75 ppm mass accuracy; 21 matched peptides; 90% coverage; MOWSE score = 103

1 MAKQDRAIRT RQTILDAAAQ VFEKQGYQAA TITEILKVAG VTKGALYFHF
51 QSKEELALGV FDAQEPPQAV PEQPLRLQEL IDMGMLFCHR LRTNVVARAG
101 VRLSMDQQAH GLDRRGPFRR WHETLLKLLN QAKENGELLP HVVTTDSADL
151 YVGTFAGIQV VSQTVSDYQD LEHRYALLQK HILPAIAVPS VLAALDLSEE
201 RGARLAAELA PTGKD

Start	End	Observed	Mr(expt)	Mr(calc)	Delta	Miss	Sequence
2	22	2372.1788	2371.1715	2371.3084	-0.1368	1	M.AKQDRAIRTRQTILDAAAQVF.E
23	47	2652.3385	2651.3312	2651.4533	-0.1221	3	F.EKQGYQAATITEILKVAGVTKGALY.F
28	47	2047.0710	2046.0638	2046.1724	-0.1086	2	Y.QAATITEILKVAGVTKGALY.F
59	75	1822.0098	1821.0025	1820.8944	0.1081	1	L.GVFDAQEPPQAVPEQPL.R
76	87	1497.6905	1496.6833	1496.7367	-0.0534	3	L.RLQELIDMGMLF.C 2-Oxidation (M)
88	103	1877.9897	1876.9825	1877.0642	-0.0817	1	F.CHRLRTNVVARAGVRL.S
92	103	1311.7528	1310.7455	1310.7894	-0.0439	0	L.RTNVVARAGVRL.S
104	118	1714.7468	1713.7395	1713.8005	-0.0610	1	L.SMDQQAHGLDRRGPF.R
104	118	1730.7224	1729.7151	1729.7954	-0.0803	1	L.SMDQQAHGLDRRGPF.R Oxidation (M)
113	118	747.3719	746.3646	746.3823	-0.0177	0	L.DRRGPF.R
127	151	2768.3271	2767.3198	2767.4391	-0.1193	4	L.KLLNQAKENGELLPHVVTTDSADLY.V
130	151	2414.0592	2413.0520	2413.1761	-0.1241	2	L.NQAKENGELLPHVVTTDSADLY.V
152	175	2712.1937	2711.1865	2711.3190	-0.1326	3	Y.VGTFAGIQVVSQTVSDYQDLEHRY.A
156	175	2307.9984	2306.9912	2307.1131	-0.1219	2	F.AGIQVVSQTVSDYQDLEHRY.A
156	178	2605.2350	2604.2277	2604.3183	-0.0906	4	F.AGIQVVSQTVSDYQDLEHRYALL.Q
176	192	1783.0211	1782.0139	1782.1131	-0.0992	2	Y.ALLQKHILPAIAVPSVL.A
179	192	1485.8562	1484.8489	1484.9078	-0.0589	0	L.QKHILPAIAVPSVL.A
193	205	1400.7096	1399.7023	1399.7419	-0.0396	2	L.AALDLSEERGARL.A
193	215	2354.1193	2353.1120	2353.2237	-0.1117	4	L.AALDLSEERGARLAAELAPTGKD.
196	215	2098.9645	2097.9573	2098.0654	-0.1081	3	L.DLSEERGARLAAELAPTGKD.
198	205	917.4419	916.4346	916.4726	-0.0380	0	L.SEERGARL.A
