# Supplementary material for: Characterisation of a natural variant of the γ-butyrolactone signalling receptor
Source: BMC Res Notes. 2012 Jul 27;5:379. doi: 10.1186/1756-0500-5-379 (PMC3461410; doi:10.1186/1756-0500-5-379)
Supplement: Additional file 2 — Primers used for (qRT-)PCR experiments. [file 1756-0500-5-379-S2.pdf]

Additional file 2

Primers used for (qRT-)PCR experiments

**Primers**	**Primer sequence (5'-3')**
actII-4rtfw	gacgcgggactggatctct
actII-4rtrv	tgcgcgatattgctttcg
BamETseq1	catggatcctcgagcagcagcatgcccggta
BamRCseq31enh	catggatccatgcgtgcacatgggacgagg
ETS10	cttcggtatccagctgaccggga
ETS10_DIG(5')	DIG- cttcggtatccagctgaccggga
ETS3	tatccagctgaccgggaacgcgtc
ETS6	atacagaacagctcggcatcac
ETS7	tgatgccgagctgttctgtatg
ETseq3	ttcggcggtcagtccttcccggtc
hrdBrtoutfw	catgcgcttcggactca
hrdBrtoutrv	actcgatctggcggatg
MalE-ScbR1	caaggacggatccatggccaagc
MalE-ScbR2	ggtgcggaagcttcggcggtcag
RCseq31	ggagcaggccggtacgtcctc
redDrtfw	tcatgggagtgcggagaac
redDrtrv	catcccccgaagttgtacag
scbArt1	tctgcgtccgatgccaactcg
scbArt2	ggtagacttgaggactggtga
scbArtfw	cgtacagggacaggtggactg
scbArtrv	cggtagacttgaggactggtgaa
scbR2	cacggcgggtcggtatccggt
scbR-M145_c358	cgaggacccttccgtc
scbR-M600_c358a	cgaggacccttccgta
ScbRrt1	caggatgtgcttctgcagcag
ScbRrt2	gcaggtcttcgagaagcaggg
scbRrtfw	tgaaccaggccaaggagaa
scbRrtrv	tctgggacacgacctgtatcc
