# Supplementary material for: Characterisation of a natural variant of the γ-butyrolactone signalling receptor
Source: BMC Res Notes. 2012 Jul 27;5:379. doi: 10.1186/1756-0500-5-379 (PMC3461410; doi:10.1186/1756-0500-5-379)
Supplement: Additional file 4 — Transcriptional analysis of redD,actII-orf4,cpkO,scbAandscbRM145/M600using qRT-PCR in GC 1.A. qRT-PCR analysis of the transcription of redD, actII-orf4, cpkO, scbA and scbRM145/M600 using cDNA synthesized from RNA isolated from liquid SMM cultures of S. coelicolor LW34 (scbRM145) and LW33 (scbRM600). Samples were taken at four time points (tp 1–4) during different phases of growth indicated with eT, mT, lT and S (early, mid, late transition, and stationary phase). Gene expression is shown as fold-change relative to the LW34 time point 1 early transition phase sample. Error bars indicate the standard deviation (see data in Additional file 3, GC 1). B. Numerical data from of the original qRT-PCR results. [file 1756-0500-5-379-S4.pdf]

Additional file 3A

Transcriptional analysis of LW34 and LW33

		Growth Curve (GC) 1				Growth Curve (GC) 2			
**time point**		**1**	**2**	**3**	**4**	**1**	**2**	**3**	**4**
**growth phase**		**eT**	**mT**	**lT**	**S**	**eT**	**mT**	**lT**	**S**
redD	**LW34**	1.00 (0.62)	5.11 (2.94)	0.34 (-0.34)	0.00 (0.00)	1.00 (0.75)	7.86 (1.53)	1.12 (0.30)	0.37 (-0.37)
	**LW33**	0.93 (0.63)	2.17 (0.79)	1.71 (0.41)	0.00 (0.00)	0.37 (-0.37)	3.16 (0.74)	2.51 (1.06)	0.44 (0.21)
actII-4	**LW34**	1.00 (0.74)	18.74 (5.79)	11.06 (2.70)	5.56 (3.16)	1.00 (0.43)	13.57 (5.46)	15.20 (7.48)	6.10 (3.95)
	**LW33**	0.43 (0.29)	26.21 (24.59)	18.41 (4.67)	1.03 (0.08)	0.34 (0.30)	2.00 (7.24)	12.85 (3.75)	3.83 (1.31)
cpkO	**LW34**	1.00 (-1.00)	15.16 (4.39)	136.68 (42.56)	18.95 (8.53)	1.00 (0.75)	7.86 (1.53)	1.12 (0.30)	0.37 (-0.37)
	**LW33**	0.00 (0.00)	0.00 (0.00)	67.29 (17.88)	7.81 (0.77)	0.23 (0.20)	2.01 (1.58)	354.15 (46.80)	36.00 (6.69)
scbA	**LW34**	1.00 (0.57)	7.86 (2.00)	7.24 (4.25)	0.23 (0.10)	1.00 (0.77)	72.05 (12.91)	8.80 (3.90)	1.04 (0.75)
	**LW33**	0.55 (0.54)	2.68 (2.09)	16.73 (3.81)	1.42 (0.60)	0.41 (0.37)	2.83 (0.34)	93.81 (53.02)	0.54 (0.44)
scbR$_{(M145/M600)}$	**LW34**	1.00 (0.46)	2.97 (0.75)	2.29 (0.30)	0.39 (0.14)	1.00 (0.30)	13.47 (2.49)	3.93 (0.37)	1.08 (0.34)
	**LW33**	0.93 (0.38)	2.96 (1.16)	5.22 (1.11)	0.49 (0.04)	0.81 (0.26)	3.62 (0.37)	10.26 (1.32)	0.90 (0.15)
