# Supplementary material for: Characterisation of a natural variant of the γ-butyrolactone signalling receptor
Source: BMC Res Notes. 2012 Jul 27;5:379. doi: 10.1186/1756-0500-5-379 (PMC3461410; doi:10.1186/1756-0500-5-379)
Supplement: Additional file 5 — Plasmids and cosmids used in this study. [file 1756-0500-5-379-S5.pdf]

Plasmids and cosmids used in this study

Name	Description	Reference
pDrive	PCR product cloning vector	Qiagen
pDrive-ScbR	pDrive harbouring *scbR*$_{M145}$	This study
pGEM-T Easy	PCR product cloning vector	Promega
pIJ2925	pUC18 derivative with a multi-cloning site flanked by *Bgl*II	[33]
pIJ6120	pIJ2925 derivative containing *scbR*$_{M145}$	[9]
pKC1132	conjugative plasmid bearing Apr^{r} *rep*^{pUC}	[21]
pSET152	*oriT attP int aac(3)*IV	[21]
pTE51	pDrive containing *scbR*$_{M600}$ with additional mutations in *scbR*$_{M600}$ (t587a) and the *scbR*$_{M600}$ promoter region (a30g in *scbA*)	This study
pTE53	pIJ2925 containing *scbR*$_{M600}$ with additional mutations in *scbR*$_{M600}$ (t587a) and the *scbR*$_{M600}$ promoter region (a30g in *scbA*)	This study
pTE56	pIJ2925 containing *scbR*$_{M600}$ with an additional mutation in the *scbR*$_{M600}$ promoter region (a30g in *scbA*)	This study
pTE58	pIJ2925 containing *scbR*$_{M600}$	This study
pTE63	pGEM-T Easy containing the *scbR*$_{M145}$ region with additional mutations in *scbR*$_{M145}$ (c636t) and *scbA* (c308t)	This study
pTE64	pGEM-T Easy containing the *scbR*$_{M600}$ region with an additional mutation in *scbA* (t77c)	This study
pTE88	pTST101 (Motejadded and Altenbuchner, 2009) with *gfp* replaced by *Bam*HI/*Hind*III *scbR*$_{M145}$ fragment from pDrive-ScbR	This study
pTE203	pGEM-T Easy containing the *scbR*$_{M600}$ region with an additional mutation in *scbB* (c644t)	This study
pTE211	pGEM-T EASY containing the *scbR*$_{M600}$ region and an additional 1·4 kb fragment containing truncated *scbA* and *scbR*$_{M600}$ genes	This study
pTE212	pKC1132 containing the *scbR*$_{M600}$ region	This study
pTE213	pGEM-T EASY containing the *scbR*$_{M145}$ region	This study
pTE214	pKC1132 containing the *scbR*$_{M145}$ region	This study
pTST101	expression plasmid containing *malE-gfp* fusion under T7and *rhaBAD* control	[27]
pUZ8002	RK2 derivative with a defective *oriT* (*aph*); not self-transmissible mobilization vector for *oriT*-containing plasmids	[34]
