# Supplementary figures and images for: Characterisation of a natural variant of the γ-butyrolactone signalling receptor
Source: BMC Res Notes. 2012 Jul 27;5:379. doi: 10.1186/1756-0500-5-379 (PMC3461410; doi:10.1186/1756-0500-5-379)

# Additional File6A

A

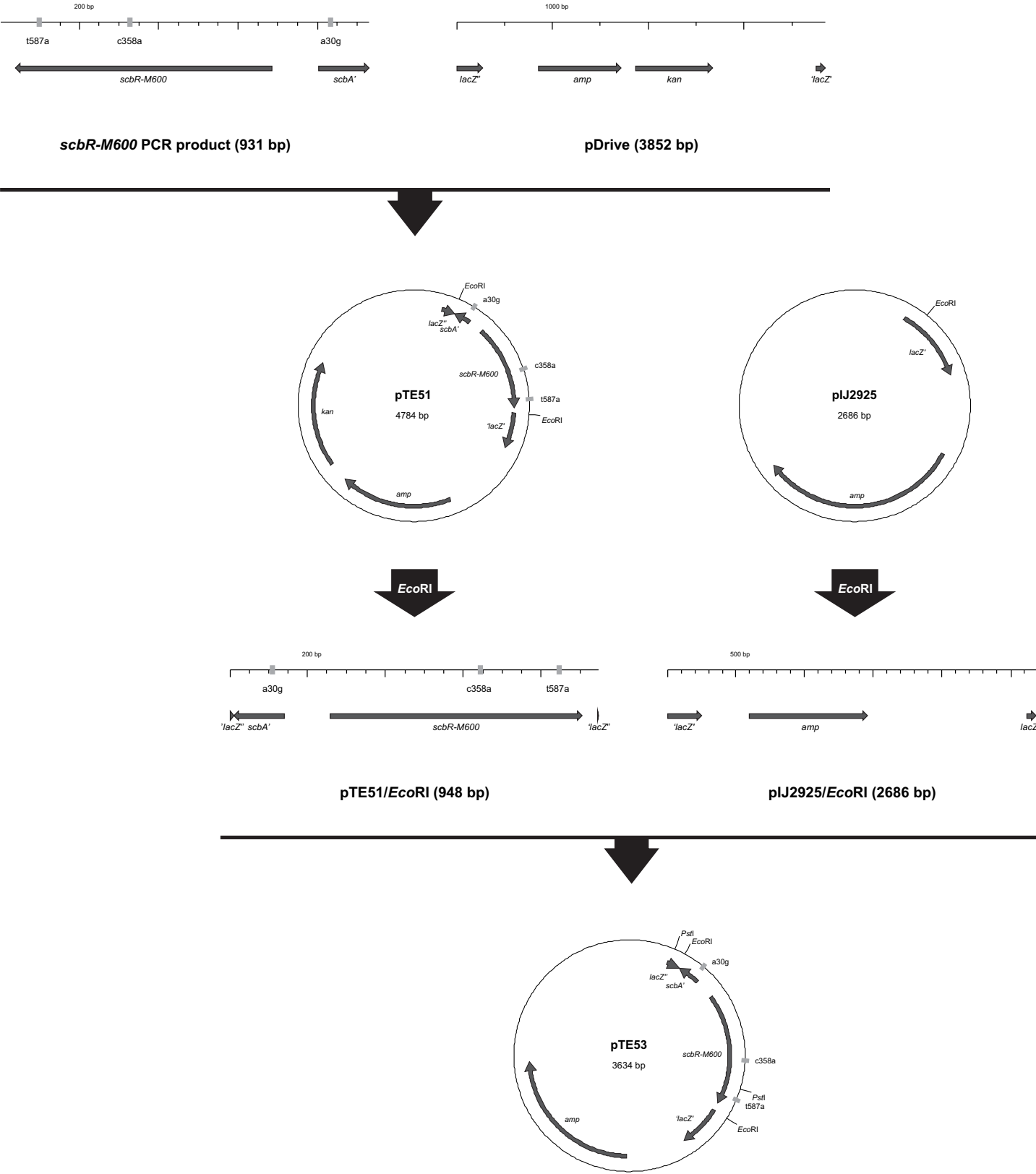

# Additional File 6B

B

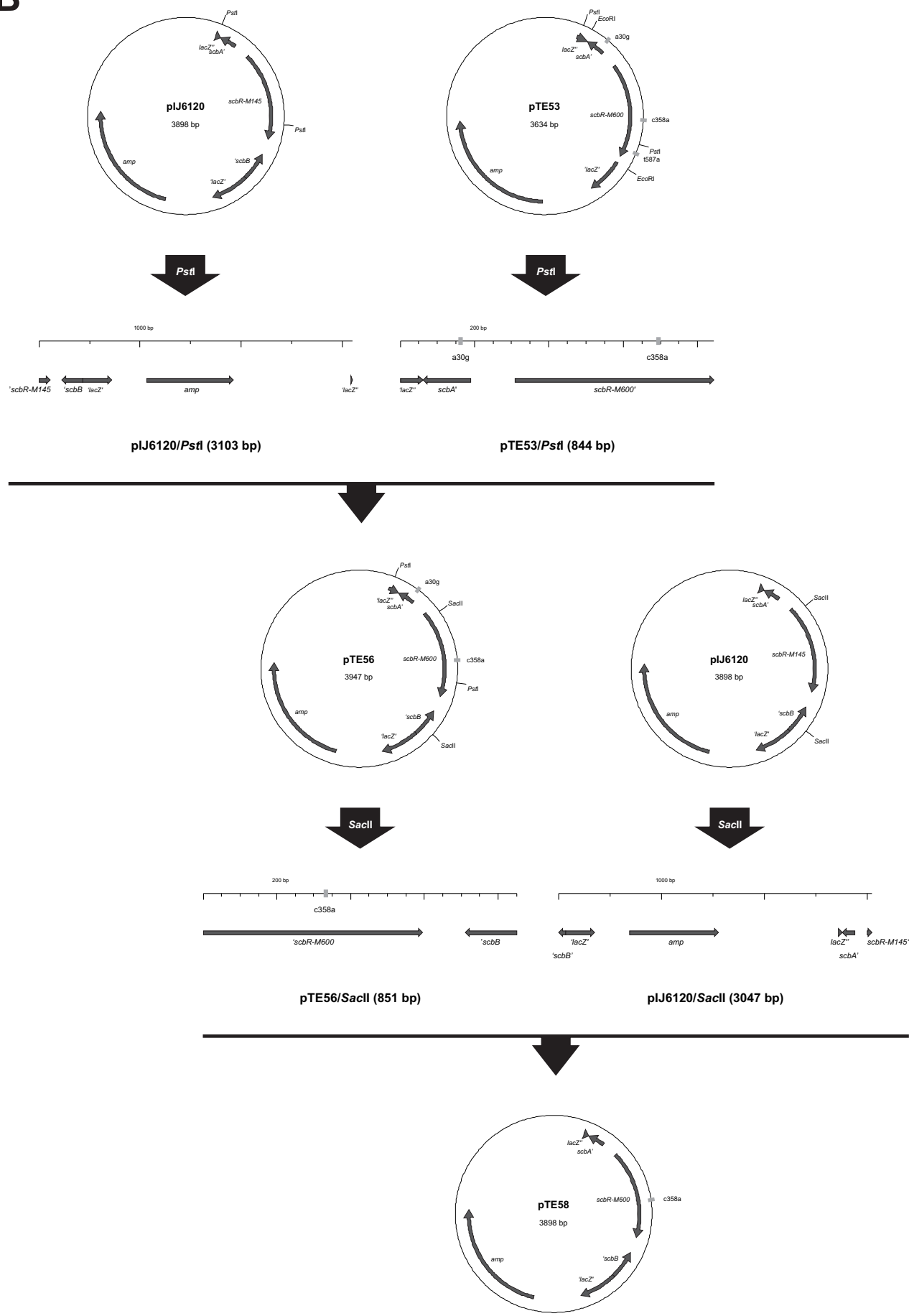

Supplement: Additional file 6 — Construction of the ScbRM600expression vector pTE58. From a genomic DNA isolate of S. coelicolorstrain M600, a 931 bp PCR fragment containing thescbRM600coding sequence and thescbRpromoter region was amplified using primers ETS3 and ScbR2 (Additional file 2). The PCR product was gel-purified and ligated to pDRIVE (Qiagen), yielding pTE51 of which the insert contains not only the natural mutation c358a in scbRM600, but also additional mutations: a30g in scbA is located in the promoter region of scbR, and nucleotide change t587a in the coding sequence of scbRM600, respectively. The point mutation t587a leads to an amino acid change, D196V, in ScbRM600 (data not shown). A 948 bp pTE51/EcoRI scbRM600fragment was cloned into the pUC18 derivate pIJ2925 to gain pTE53 containing all three described mutations (A). A 844 bp pTE53/PstI fragment containing the scbR promoter region and nucleotides 1-535 of the scbRM600coding region was ligated into a 3103 bp pIJ6120/PstI vector fragment [9] containing nucleotides 536–648 of the scbRM145coding region, yielding pTE56. Partial sequence analysis of pTE56 revealed only the scbR promoter mutation a30g in scbA and the expected coding sequence mutation c358a of scbRM600. A 851 bp pTE56/SacII fragment containing nucleotides 54–304 of scbRM600was cloned into a 3047 bp pIJ6120/SacII vector fragment containing the scbR promoter region and nucleotides 1–53 and 305–648 of the scbRM145coding sequence, yielding pTE58 with scbR M600 and the scbR promoter region without additional undesired mutations in the same orientation as the IPTG-inducible E. colilacZ promoter (B). The scbRM145 expression construct pIJ6120 [9] and pTE58 differ only by the natural mutation, c358a, in scbRM600 and were used for the heterologous expression of ScbRM145 and ScbRM600 in E. coli. Linear DNA fragments are named and a scale is given with indicated base pair (bp) units. Plasmids are named and represented by black circles. Genes are indicated by dark arrows and labell [file 1756-0500-5-379-S6.pdf]

# Additional File 7A,B,C

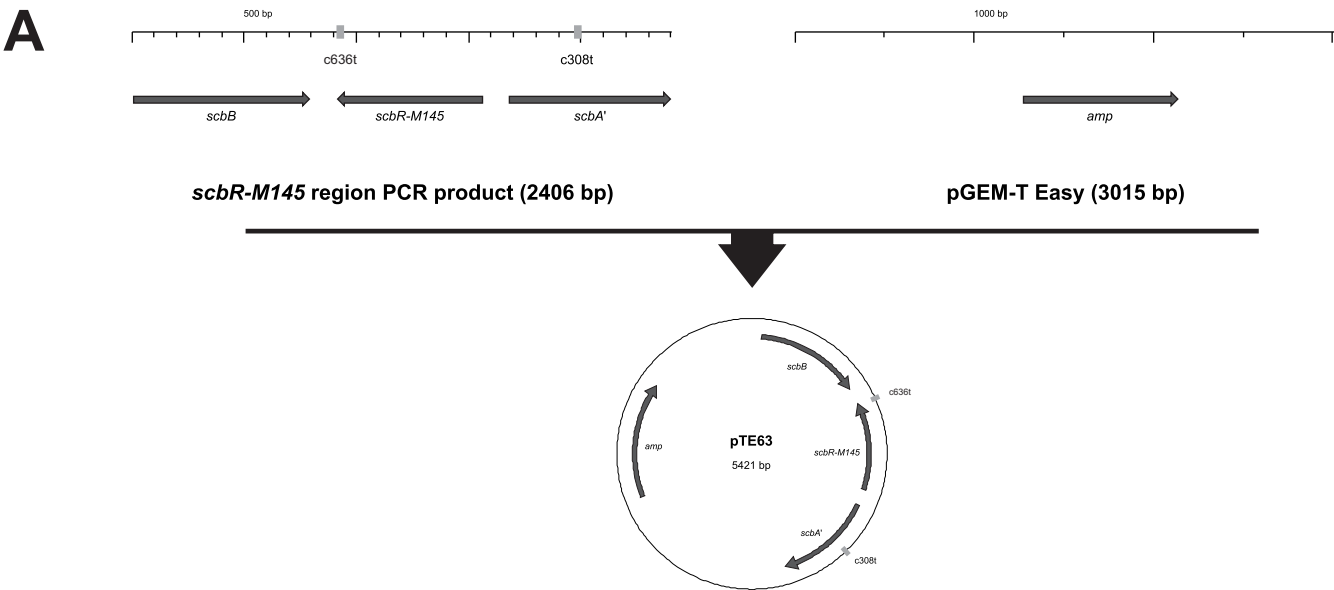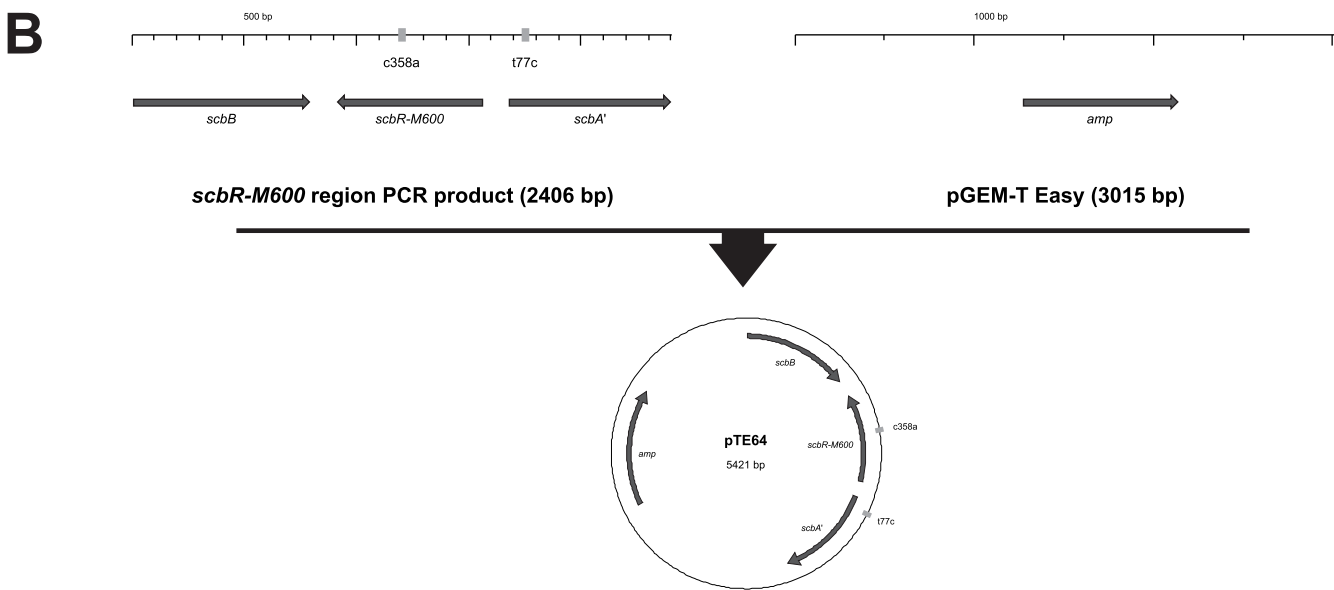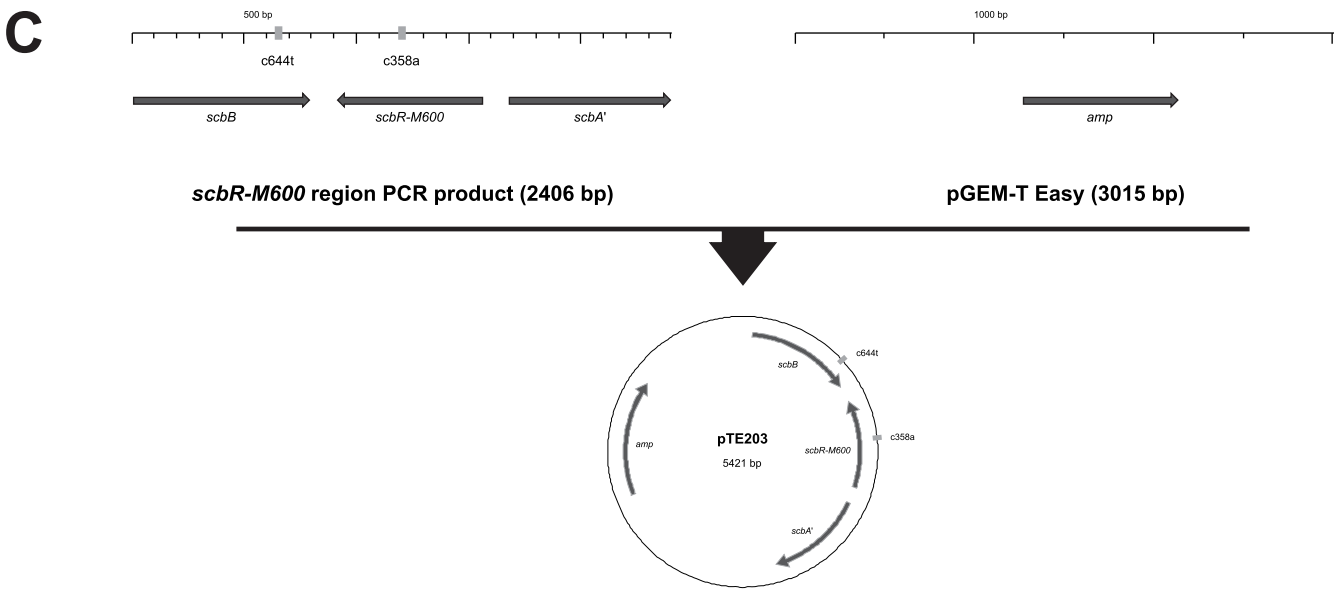

# Additional File 7E

E

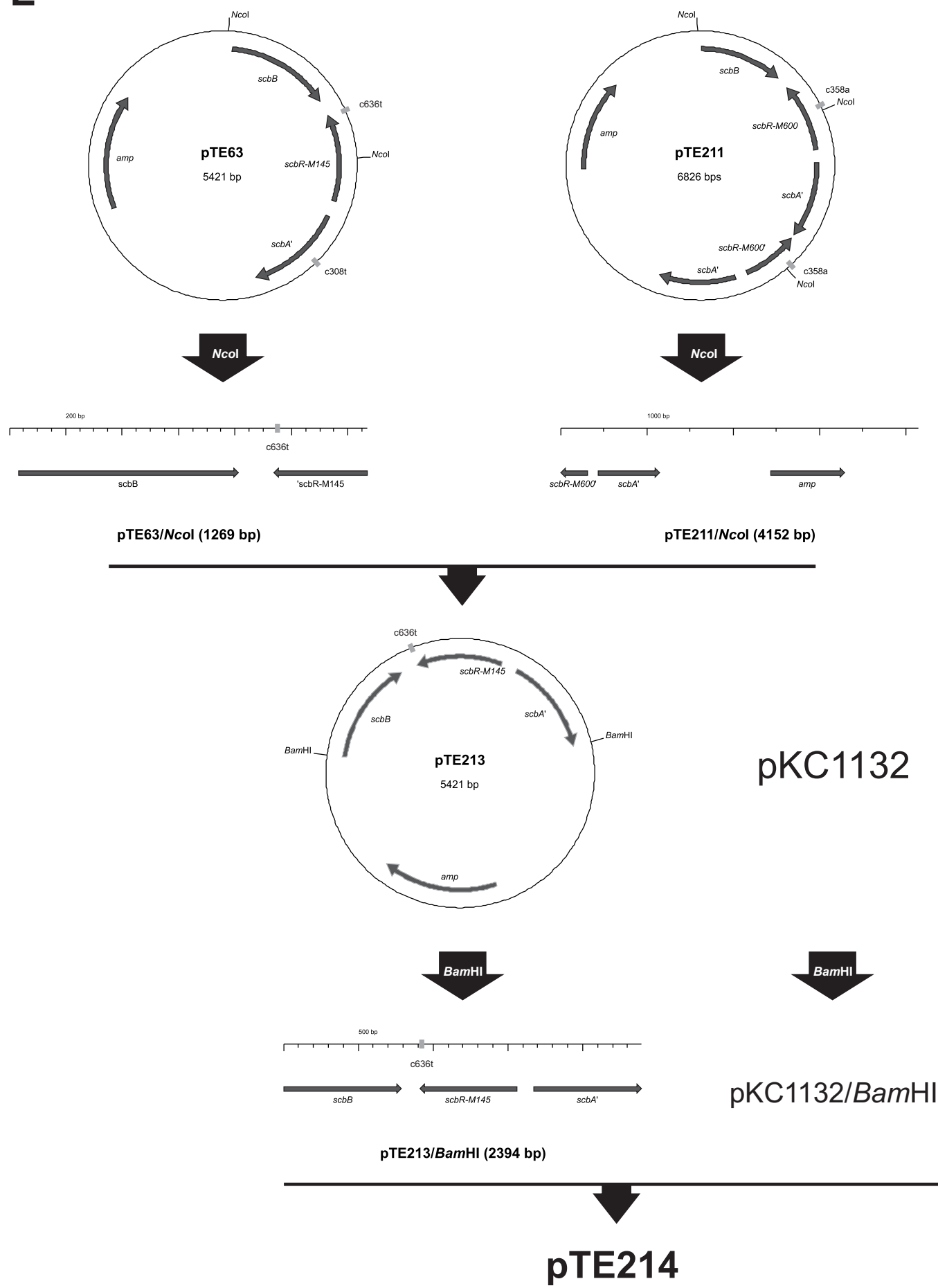

# Additional File 7D

D

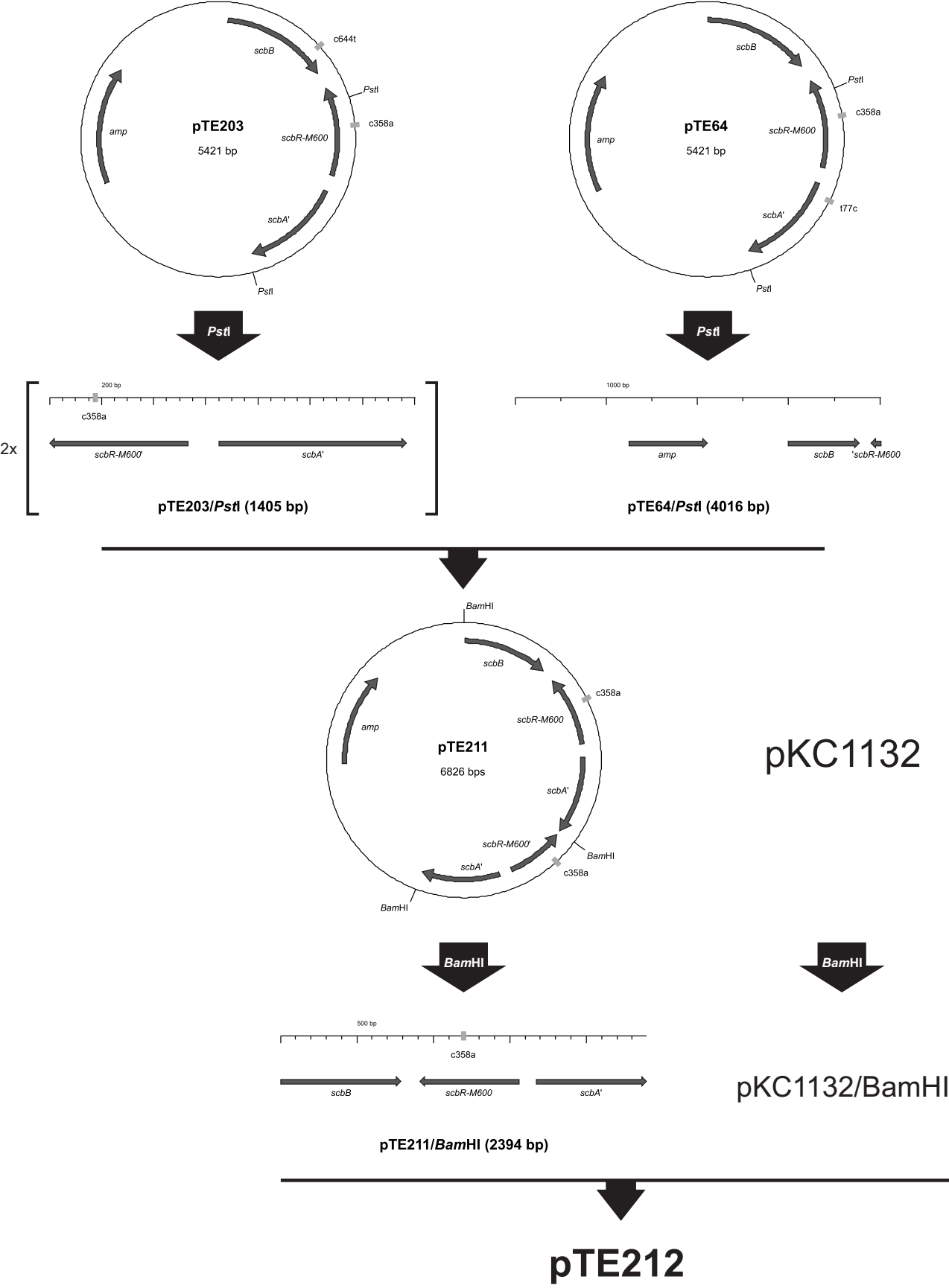

Supplement: Additional file 7 — Construction of the complementation vectors pTE212 and pTE214. From genomic DNA isolates of S. coelicolor strains M145 and M600, a 2406 bp PCR fragment containing the scbRM145/M600 coding sequence and flanking regions was amplified using primers BamRCseq31enh and BamETseq1 (Additional file 2). PCR products were gel-purified and ligated to pGEM-T EASY (Promega), yielding pTE63 harbouring scbRM145 (A), and pTE64 (B) and pTE203 (C) harbouring scbRM600. The inserts of the plasmids were sequenced and pTE63 was found to contain mutations in scbRM145 (silent mutation c636t) and scbA (c308t leading to A103V). pTE64 contains a mutation in scbA (t77c leading to M26T), and pTE203 in scbB (c644t leading to A215V). Two 1405 bp pTE203/PstI fragments were introduced by tandem integration into a 4016 bp pTE64/PstI vector fragment to give pTE211, which is pGEM-T EASY with a 2394 bp BamHI scbRM600fragment without any additional mutations and an additional 1.4 kb BamHI fragment. The 2394 bp pTE211/BamHI fragment was cloned into pKC1132 to give pTE212 (D). A 1269 bp pTE63/NcoI fragment containing the silent mutation c636t in scbRM145 was cloned into a 4152 bp pTE211/NcoI vector fragment to give pTE213, which is pGEM-T EASY with a 2394 bp BamHI scbRM145fragment with only the silent mutation. The 2394 bp pTE213/BamHI fragment was cloned into pKC1132 to give pTE214 (E). Linear DNA fragments are named and a scale is given with indicated base pair (bp) units. Plasmids are named and represented by black circles. Genes are indicated by dark arrows and labelled with gene names. For incomplete genes the missing part is indicated by an apostrophe at the beginning or the end of the gene name. The ampicillin antibiotic resistance gene is abbreviated with “amp”. The position of the described mutations is indicated by labelled grey boxes on the scales and in the plasmid maps; relevant restriction sites are shown with enzyme names. Thick black lines with arrows indicate ligation events; big black arr [file 1756-0500-5-379-S7.pdf]
